# Shared genetic architecture and causal relationship between sleep behaviors and lifespan

**DOI:** 10.1038/s41398-024-02826-x

**Published:** 2024-02-22

**Authors:** Yong Wu, Chu-Yi Zhang, Xiaolan Liu, Lu Wang, Ming Li, Yi Li, Xiao Xiao

**Affiliations:** 1 Research Center for Mental Health and Neuroscience, Wuhan Mental Health Center, Wuhan, Hubei China; 2 Affiliated Wuhan Mental Health Center, Jianghan University, Wuhan, Hubei China; 3 Yunnan Key Laboratory of Animal Models and Human Disease Mechanisms, Kunming Institute of Zoology, Chinese Academy of Sciences, Kunming, Yunnan China; 4 Kunming College of Life Science, University of Chinese Academy of Sciences, Kunming, Yunnan China; 5 Research Center for Psychological and Health Sciences, China University of Geosciences, Wuhan, Hubei China

**Keywords:** Clinical genetics, Diseases

## Abstract

Poor sleep health is associated with a wide array of increased risk for cardiovascular, metabolic and mental health problems as well as all-cause mortality in observational studies, suggesting potential links between sleep health and lifespan. However, it has yet to be determined whether sleep health is genetically or/and causally associated with lifespan. In this study, we firstly studied the genome-wide genetic association between four sleep behaviors (short sleep duration, long sleep duration, insomnia, and sleep chronotype) and lifespan using GWAS summary statistics, and both sleep duration time and insomnia were negatively correlated with lifespan. Then, two-sample Mendelian randomization (MR) and multivariable MR analyses were applied to explore the causal effects between sleep behaviors and lifespan. We found that genetically predicted short sleep duration was causally and negatively associated with lifespan in univariable and multivariable MR analyses, and this effect was partially mediated by coronary artery disease (CAD), type 2 diabetes (T2D) and depression. In contrast, we found that insomnia had no causal effects on lifespan. Our results further confirmed the negative effects of short sleep duration on lifespan and suggested that extension of sleep may benefit the physical health of individuals with sleep loss. Further attention should be given to such public health issues.

## Introduction

Human lifespan is impacted by many factors, including genetics, health, disease, lifestyle, environment, and chance. The estimated genetic heritability of human lifespan ranges from ~16% based on population data to ~25% based on twin studies [1–5], and many genetic studies have been conducted to identify certain associated variables. For example, genome-wide association studies (GWASs) have compared the differences in common variants between individuals who live longer (i.e., longevity) and controls [6–9]. Some studies also considered lifespan as a quantitative trait in the general population and used survival models (such as Cox proportional hazards) to allow long-lived survivors to inform analysis [10]. Both approaches have revealed useful information for understanding the genetic basis of lifespan.

Human sleep can be characterized by duration, which was measured as sleep time in one day, also known as sleep chronotype. There are morning people (or “larks”, referring to people who prefer going to bed and waking earlier) or evening people (or “owls”, referring to people who prefer a later bedtime and later rising time) according to the sleep habits. Efficiency and regularity, sleep-related behaviors and sleep disorders (most commonly seen as insomnia, which was characterized by difficulty in falling or remaining asleep) are heritable complex traits, with estimated genetic heritability ranging 9 ~ 45% based on twin and family studies [11–14]. Mounting data have shown that poor sleep health is a potentially modifiable risk factor for cardiovascular, metabolic and mental health problems as well as all-cause mortality [15–22], suggesting potential links between poor sleep health and lifespan. However, it is also acknowledged that traditional observational studies may be biased by multiple residual confounding effects; for example, low socioeconomic status and poor general health may confound the observed correlations between poor sleep health and cardiovascular diseases [23]. It is, therefore, yet to be determined whether sleep health and lifespan have any causal relationship to each other or whether their epidemiological associations are because of the common risk factors with horizontal pleiotropy.

For causation across different complex traits, Mendelian randomization (MR) can be used to infer potential causal relationships based on SNP statistics in GWAS [24, 25]. Single nucleotide polymorphisms (SNPs) were used to calculate causality because they were randomly allocated at meiosis and fertilization before birth and were unlikely to be influenced by disease status, environmental stimuli or other confounding factors [26] and, thus, may reflect more authentic causal associations compared to traditional observational studies. Notably, MR has been extensively used to evaluate the causal associations between sleep-related behaviors and many complex traits, such as psychiatric conditions and cardiovascular diseases [27–30].

Here, to assess the genetic architecture and causal relationship between sleep behaviors and lifespan, we calculated the genetic correlation and estimated the polygenic overlap between sleep behaviors and lifespan using the GWAS summary statistics. We then inferred the potential causal relationship from sleep behaviors to lifespan using two-sample MR and multivariable MR analyses. We also conducted a two-step MR analysis to assess the mediation effects of poor sleep health on lifespan via coronary artery disease (CAD), ischemic stroke (AIS), type 2 diabetes (T2D), psychiatric disorders, heart failure, atrial fibrillation and body mass index (BMI).

## Methods

### GWAS data of sleep-related behaviors

Regarding sleep duration, participants were asked about how many hours sleep they had in every 24 h (including naps), with responses in hour increments. European ancestry GWASs were conducted separately for duration short (<7 h per night, *n* = 106,192 cases) and duration long (>9 h per night, *n* = 34,184 cases) relative to sleep duration >7 h and <9 h (*n* = 305,742 controls) [31]. A total of 27 and 8 loci (*P* < 5.00 × 10^–8^) were identified as being associated with duration short and duration long, respectively. GWAS summary statistics can be downloaded from the Sleep Disorder Knowledge Portal at https://sleep.hugeamp.org/downloads.html.

For sleep chronotype, participants were required to answer the question “Do you consider yourself to be?” with one of six possible answers as follows: “Definitely a ‘morning’ person”, “More a ‘morning’ than ‘evening’ person”, “More an ‘evening’ than a ‘morning’ person”, “Definitely an ‘evening’ person”, “Do not know” or “Prefer not to answer”. Participants answering “Definitely a ‘morning’ person” and “more a ‘morning’ than an ‘evening’ person” were defined as cases. Participants answering “Definitely an ‘evening’ person” and “more an ‘evening’ than a ‘morning’ person” were defined as controls. A total of 252,287 cases and 150,908 controls from European ancestry were included in the GWAS conducted by Jones et al. [32]. GWAS summary statistics can also be downloaded from the Sleep Disorder Knowledge Portal.

For insomnia, participants were asked a single question: “Do you have trouble falling asleep at night or do you wake up in the middle of the night?” Four answer possibilities were supplied: “never/rarely”, “sometimes”, “usually”, or “prefer not to answer”. Participants who answered “usually” were defined as insomnia cases, and those who answered “never/rarely” were defined as controls. A total of 66,976 cases and 141,982 controls from European ancestry were included in the GWAS conducted by Watanabe et al. [33]. GWAS summary statistics can be downloaded from https://ctg.cncr.nl/software/summary_statistics/.

### Lifespan GWAS data

Timmers et al. used data from the UK Biobank and 26 independent European-heritage population cohorts to conduct a GWAS of parental survival, quantified using Cox models [10]. Briefly, association test between parental survival (age and alive/dead status) and offspring genotype was conducted using Cox Proportional Hazards Model [6]. Survival traits were transformed into residuals, where higher values indicate longer life, to make lifespan as quantitative traits. A sample of 1,012,240 parents (60% deceased) was meta-analyzed, and 12 genomic regions were found to be significantly associated with lifespan. GWAS summary statistics can be downloaded at 10.7488/ds/2463.

### Other GWAS data

We included GWAS data of CAD [34], AIS regardless of subtype [35], T2D (adjusted for BMI) [36], heart failure [37], atrial fibrillation [38] and BMI [39], depression [40], bipolar disorder (BD) [41], schizophrenia (SZ) [42] in our analysis. The sample size of these studies ranged from 130,644 (SZ) to 964,057 (heart failure). All these GWASs, except for atrial fibrillation, used samples exclusively from European ancestry. In the atrial fibrillation GWAS, a total of 65,446 cases and 522,744 controls were included, among which 55,114 cases and 482,295 controls represented European ancestry, thus accounting for 84.2% of the total samples [38]. The detailed information of all these GWASs, including sample size, ancestry, and publication, is summarized in Supplementary Table S1.

### Genome-wide genetic correlation estimation

We used linkage disequilibrium score regression (LDSC, v1.0.01) to estimate the genome-wide genetic correlation between sleep behaviors and lifespan [43]. Genome-wide genetic correlation could help us to understand the genetic relationship between different traits. The LDSC estimated genetics correlation from summary statistics based on the fact that the heritability of complex traits is distributed over thousands of variants with small effects. The GWAS summary statistics data was first reformatted and then used to compute the genetic correlation. Genotype information of European ancestry from 1000 genomes [44] were used as reference panel during LDSC analysis.

### Polygenic variants estimation

The bivariate causal mixture model (MiXeR, v1.3) was used to estimate the total number of shared and trait-specific genetic variants between sleep behaviors and lifespan [45]. MiXeR firstly estimated the total amount of causal variants of each trait based on the univariate causal mixture model and then intersected the causal variants of two traits using the bivariate normal distribution. The results of MiXeR were illustrated with Venn plot to show the number of shared and unique variants of two traits. The GWAS summary statistics data was firstly pre-processed to calculate the Z-score and remove variants in major histocompatibility complex (MHC) region (hg19, chr6: 26-34 Mb) due to the complex linkage disequilibrium (LD) of this region [46]. Then, the univariate analysis of MiXeR was applied to estimate the number of causal variants of one trait and the bivariate (cross-trait) analysis was sequentially applied to estimate the number of shared variants between two traits. Genotype information of European ancestry from 1000 genomes [44] were used as reference panel during MiXeR analysis.

### Shared genetic loci identification

We next applied the conjunctional false discovery rate (conjFDR) approach to identify the shared genetic loci [47]. This approach builds on an empirical Bayesian statistical framework to uncover overlapping genetic variants irrespective of direction of effects. Unlike MiXeR, the conjFDR identifies the specific shared loci between two traits, and thus offer genetic overlap on different levels. The summary statistics data was firstly pre-processed and then the conjFDR was run on the GWAS data with default parameters. According to the suggestion from the author of conjFDR, genetic loci at conjFDR <0.05 were considered as shared loci.

### Genetic instrument variables

MR used genetic instrument variables (IVs) to infer the causality between exposures and outcomes, so the choice of IVs is crucial. We conducted a series of quality controls to select eligible IVs. Firstly, according to the first basic assumptions of MR, that is the genetic variants should be strongly associated with exposures [26, 48], we extracted independent SNPs associated with exposures at genome-wide significance (*P* < 5 × 10^–8^) using clumping method in PLINK (Version 1.9) [49]. We set LD clumping with r^2^ < 0.001, *P* < 5 × 10^–8^ and a window size of 10 Mb to select independent significant SNPs. As most GWAS samples were of Europeans, we used the genome data of European ancestry from 1000 genomes clumping reference panel [44]. Secondly, we extracted the GWAS summary statistics of IVs in outcome GWAS data. If a particular IV was missed in outcome GWAS, we used a proxy SNP in high LD (r^2^ > 0.8) with the requested SNP for instead. We searched the proxy SNP in SNiPA (https://snipa.helmholtz-muenchen.de/snipa3/index.php) [50]. Thirdly, the returned SNPs were harmonized to ensure that the effect alleles of potential instrumental SNPs in exposure and outcome GWASs were aligned to the same genome strand. Palindromic SNPs with ambiguous strands (i.e., A/T or G/C) and intermediate allele frequencies (>0.42) were removed. Lastly, the MR-Pleiotropy RESidual Sum and Outlier (MR-PRESSO) method were used to detect and remove outlier SNPs [51]. We set the number of distributions in MR-PRESSO analysis as 1000. SNPs with *P* value < 0.05 in MR-PRESSO outlier test were removed to reduce heterogeneity. The remained SNPs were used as bona fide IVs for further two-sample MR analysis. To test whether there are weak instrumental variables, we calculated the *F* statistics, which is a measure of instrument strength, of exposure GWAS using the following formula: *F* = *R*^2^(*N* – *k* - 1)/*k*(1 − *R*^2^) [52], of which *R*^2^ is the variance of exposure explained by selected instrumental variables; *N* is the total sample size of exposure GWAS and k is the number of IV. We got *R*^2^ from MR Steiger directionality test [53]. If the *F* statistics larger than 10, the possibility of weak instrumental variable bias is small [54].

### Two-sample MR study design

We further used a two-sample MR strategy to infer the causality between sleep behaviors and lifespan with the TwosampleMR (version 0.5.6) package [53, 55]. We leveraged inverse variance weighting (IVW), which uses a meta-analysis approach to combine Wald estimates for each SNP to obtain the overall estimates of the effect of exposure on outcome [56], as the primary MR analysis method. This approach assumed that all the included variants were valid or horizontal pleiotropy-balanced IVs that did not violate the three basic assumptions of MR. Other methods, including MR-Egger [57], weighted median [58], weighted mode [59] and robust adjusted profile score (MR.RAPS) [60], were used as auxiliary methods to estimate the causal effect. Briefly, MR-Egger detects small study bias in meta-analysis by conducting a weighted linear regression of the outcome coefficients on the exposure coefficients and can give a valid and consistent causal effect estimate even when all the genetic variants are invalid IVs. However, this approach is substantially less efficient than IVW and median-based methods and is likely to be imprecise if all genetic variants have a similar effect on exposure [58]. The weighted median method combines data on multiple genetic variants into a single causal estimate and allows up to 50% of the information to come from invalid IVs [58]. The mode-based methods cluster the SNPs into groups based on their similarity of causal effects and estimate the causal effect according to the clusters that have the largest number of SNPs [59]. This method has less bias and lower type-I error rates than other methods under the condition that the SNPs contributing to the largest cluster are valid IVs even if the majority of IVs are invalid [59]. MR.RAPS estimates the causal effects based on a robust adjusted profile scores function and is robust to both systematic and idiosyncratic pleiotropy, especially when the exposure and the outcome are both complex traits [60].

We performed a series of sensitivity analyses to determine potential bias and make our results more reliable. First, since the IVW estimates could be biased due to the heterogeneity of SNP effect size, we calculated Cochran’s Q statistics to evaluate heterogeneity in instrument effects [61]. A *P*-value of the Cochran’s Q test larger than 0.05 indicated that the effect of heterogeneity was small. If heterogeneity existed, we applied the random-effect model of IVW to estimate the causal effect. Second, the widespread SNP horizontal pleiotropy effects could result in violations of Mendel’s Third Law of exclusion restriction assumption, that is, the IVs could not directly influence the outcome except through exposure [48, 62]. We assessed the potential pleiotropic effects of IVs by comparing the difference between the MR-Egger regression intercept term and zero. No significant difference between the intercept term of MR-Egger regression and zero (*P* > 0.05) indicated no horizontal pleiotropy [63]. Third, we performed an MR Steiger directional test to determine whether the assumption that exposure causes outcome is valid [53]. If the *R*^2^ (same as *R*^2^ in *F* statistics, which means the variances of exposure or outcome explained by the selected IVs) of outcome was significantly less than that of exposure (MR Steiger test *P* < 0.05), we could say no reverse causality existed between exposure and outcome. Finally, leave-one-out analysis was used to test whether the causal effect was robust. This method removes one IV each time and conducts IVW analysis using the rest of the IVs to assess the influence of individual variants on the causal effect.

### Multivariable MR

As sleep behaviors, sleep duration time, sleep chronotype and insomnia, were related to each other, and each of the three traits could be a confounder to the other remaining traits when conducting univariable or standard MR [64]. Thus, the univariable MR analysis of any of these sleep behaviors on lifespan may potentially be biased by pleiotropic effects through the other sleep behaviors. Considering this, we performed multivariable MR (MVMR) analysis, which uses a formula of pleiotropy adjustment by including all of these sleep behaviors in the same model. We combined all the variants included in the univariable MR of sleep behaviors on lifespan as IVs in MVMR analysis. The “MendelianRandomization” R package (version 0.6.0, https://github.com/cran/MendelianRandomization) was used to conduct MVMR analysis. The IVW and MR-Egger methods were used to estimate the causal effect of one sleep behavior condition on the other two sleep behaviors. We tested for heterogeneity in the SNP outcome association by calculating the adjusted Cochran Q statistic with summary data [65]. The pleiotropy effect was also evaluated by testing the difference between the MR-Egger intercept term and zero. We used IVW as the primary method to estimate the MVMR effects under the conditions of no pleiotropy effect. Otherwise, the multivariable MR-Egger, which allows for directional pleiotropy, was used to estimate the causal effects [66].

### Mediation analysis

Finally, we tried to identify the mediators of the causal relationship between sleep behaviors and lifespan by mediation analysis, which is a field of analysis that attempts to find the causal pathways by which exposure influences an outcome [64]. Generally, mediation analysis is a two-step MR analysis that first assesses the causal relationship between exposure and mediator and then determines the causality between mediator and outcome. In the first step, genetic variants significantly associated with exposure were used as the instrumental variables to infer the causality between short sleep duration and potential mediators. Then, mediators significantly associated with exposures were further used as exposures to infer the causalities between mediators and lifespan. The product of coefficients method was used to estimate the indirect effect by multiplying the effect of exposure on mediator and the effect of mediator on outcome [64].

### Ethics and consent

All the GWAS data used in this study were publicly available and no original data was collected. Each included study was approved by their institutional ethics review committees, and informed consents (Consent to Participate and Consent to Publish) were obtained from all participants.

## Results

### Genetics correlation

**Fig. 1:**
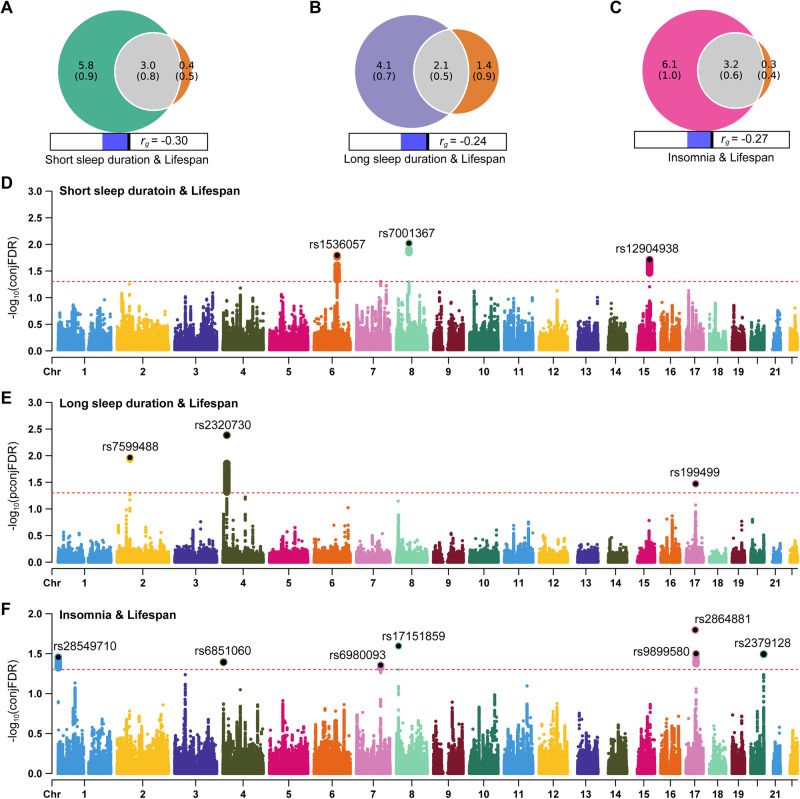
Polygenic overlap and shared genetic loci between sleep-related behaviors and lifespan. **A–C** Venn diagrams of MiXeR show the unique and shared variants associated with short sleep duration, long sleep duration, insomnia and lifespan. The numbers in each component indicate the estimated number of variants in thousands with standard deviations in parentheses. **D–F** Manhattan plots of conjFDR show the specific shared loci between sleep-related loci and lifespan. The red dotted line represents the conjFDR threshold for significant association <0.05. The flag SNPs in shared loci are marked with black dot and text.

We first used the LDSC to calculate the genome-wide genetic correlation between sleep behaviors and lifespan. The LDSC results showed that both short sleep duration (*r*_*g*_ = –0.30, *P* = 1.27 × 10^–15^), long sleep duration (*r*_*g*_ = –0.24, *P* = 5.50 × 10^–9^) and insomnia (*r*_*g*_ = –0.27, *P* = 2.11 × 10^–13^) were significantly negatively correlated with lifespan. No significant genetic correlation was found between sleep chronotype and lifespan (*r*_*g*_ = –0.05, *P* = 0.09). Univariate MiXeR estimated that sleep behaviors were more polygenic relative to lifespan, as 8800, 6200 and 9300 variants estimated to influence short sleep duration, long sleep duration and insomnia, respectively, whereas only ~3500 variants influenced lifespan (Fig. 1A–C). Bivariate MiXeR revealed that about 3000, 2100 and 3200 variants were shared between short sleep duration & lifespan, long sleep duration & lifespan and insomnia & lifespan, accounting for more 60% of the total variants to lifespan, and even about 90% of the total variants to lifespan were shared between short sleep duration & lifespan and insomnia & lifespan (Fig. 1A–C). We further applied conjFDR to identify the shared loci between sleep behaviors and lifespan. There were 3, 3, and 7 loci respectively shared between short sleep duration & lifespan, long sleep duration & lifespan and insomnia & lifespan (Fig. 1D–F). All these results suggested that sleep behaviors and lifespan were genetically correlated.

### Univariable MR

**Fig. 2:**
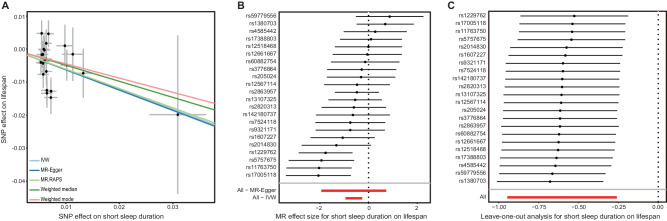
Causal relationship of short sleep duration on lifespan. **A** Scatter plot of two sample MR results of short sleep duration on lifespan. The Slop of different colorful lines represent the estimated MR effect of different MR methods. **B** Forest plot of short sleep duration on lifespan. Each line indicates the effect of an IV. **C** Forest plot of leave-one-out result of short sleep duration on lifespan. Each line shows the result of IVW estimate of short sleep duration on lifespan after removing this SNP. No effect line crosses zero indicates the result is robust.

To investigate the relationship between lifespan and sleep behaviors (including sleep duration time, insomnia, and sleep chronotype), we designed a two-sample MR strategy. The significant and independent genetic variants associated with sleep behaviors were used as instrumental variables to infer causality (Supplementary Table S2). The results showed that short sleep duration had a negative causal effect on lifespan (IVW: *β* = –0.60, 95% confidence interval (CI) = –0.95 to –0.26, *P* = 5.78 × 10^–4^, Table 1), which can be interpreted to mean that every s.d. increase in the tendency for a short sleep duration causes a loss in lifespan of 0.6 years. The other four MR methods also gave consistent estimates (weighted median: *β* = –0.49, 95%CI = –0.90 to –0.07, *P* = 0.02; MR-Egger: *β* = –0.61, 95%CI = –1.94 to 0.73, *P* = 0.38; weighted mode: *β* = –0.43, 95%CI = –1.15 to 0.29, *P* = 0.25; MR.RAPS: *β* = –0.59, 95%CI = –0.96 to –0.22, *P* = 1.79 × 10^–3^, Table 1), further supporting the result that short sleep duration was a risk factor for reduced lifespan. The results of the MR-Egger and weighted mode methods failed to pass the significance test, which might be because the capability of these two methods was less than that of the other three methods [59]. We also present the MR results in scatter plots with different colors for the tendency lines to indicate estimates from the different methods (Fig. 2A) and forest plots with the causal effect of each IV of short sleep duration on lifespan (Fig. 2B). As the result of MR is susceptible to pleiotropy and the effect of a single IV, we conducted a series of additional analyses to determine potential bias. Cohran’s Q statistic indicated no heterogeneity across IVs (IVW: Q = 32.6, df = 21, *P* = 0.051). No significant evidence of horizontal pleiotropy was determined as indicated by the MR-Egger regression intercept term being close to zero with *P* = 0.997. The leave-one-out test result showed that the causal effect was not driven by any one of the IVs used (Fig. 2C). The causal direction from exposure to outcome was also valid (exposure SNP r^2^: 2.01 × 10^–3^; outcome SNP r^2^: 8.07 × 10^–5^; MR Steiger *P* = 1.41 × 10^–71^).

**Table 1:** Univariable MR results of sleep duration, sleep chronotype and insomnia on lifespan.

MR Method	Number of IVs	*F*	*β* (95%CI)	*P*
**Short sleep duration on lifespan**				
IVW	22	38	–0.60 (–0.95 to –0.26)	5.78E–04
Weighted median			–0.49 (–0.90 to –0.07)	0.02
MR-Egger			–0.61 (–1.94 to 0.73)	0.38
Weighted mode			–0.43 (–1.15 to 0.29)	0.25
MR.RAPS			–0.59 (–0.96 to –0.22)	1.79E–03
**Long sleep duration on lifespan**				
IVW	5	39	0.24 (–0.52 to 1.00)	0.54
Weighted median			0.30 (–0.68 to 1.28)	0.54
MR-Egger			1.34 (–3.71 to 1.02)	0.35
Weighted mode			0.69 (–0.85 to 2.23)	0.43
MR.RAPS			0.25 (–0.55 to 1.05)	0.55
**Sleep chronotype on lifespan**				
IVW	113	45	0.06 (–0.06 to 0.19)	0.34
Weighted median			0.10 (–0.07 to 0.26)	0.24
MR-Egger			0.15 (–0.26 to 0.56)	0.47
Weighted mode			0.22 (–0.15 to 0.59)	0.25
MR.RAPS			0.07 (–0.06 to 0.20)	0.32
**Insomnia on lifespan**				
IVW	11	43	–0.01 (–0.10 to 0.08)	0.83
Weighted median			0.03 (–0.08 to 0.13)	0.62
MR-Egger			0.09 (–0.18 to 0.37)	0.52
Weighted mode			–0.06 (–0.09 to 0.21)	0.46
MR.RAPS			0.0008 (–0.09 to 0.09)	0.99

No causal effects were determined from long sleep duration time (IVW: *β* = 0.24, 95%CI = –0.52 to 1.00, *P* = 0.54; weighted median: *β* = 0.30, 95%CI = –0.68 to 1.28, *P* = 0.54; MR-Egger: *β* = 1.34, 95%CI = –3.71 to 1.02, *P* = 0.35; weighted mode: *β* = 0.69, 95%CI = –0.85 to 2.23, *P* = 0.43; MR.RAPS: *β* = 0.25, 95%CI = –0.55 to 1.05, *P* = 0.55), sleep chronotype (IVW: *β* = 0.06, 95%CI = –0.06 to 0.19, *P* = 0.34; weighted median: *β* = 0.10, 95%CI = –0.07 to 0.26, *P* = 0.24; MR-Egger: *β* = 0.15, 95%CI = –0.26 to 0.56, *P* = 0.47; weighted mode: *β* = 0.22, 95%CI = –0.15 to 0.59, *P* = 0.25; MR.RAPS: *β* = 0.07, 95%CI = –0.06 to 0.20, *P* = 0.32), and insomnia to lifespan (IVW: *β* = –0.01, 95%CI = –0.10 to 0.08, *P* = 0.83; weighted median: *β* = 0.03, 95%CI = –0.08 to 0.13, *P* = 0.62; MR-Egger: *β* = 0.09, 95%CI = –0.18 to 0.37, *P* = 0.52; weighted mode: *β* = –0.06, 95%CI = –0.09 to 0.21, *P* = 0.46; MR.RAPS: *β* = 0.0008, 95%CI = –0.09 to 0.09, *P* = 0.99) (Table 1 and Supplementary Figure S1).

### Multivariable MR

As sleep duration time, insomnia and sleep chronotype were related to each other, the univariable MR analysis of one of these traits could not rule out confounding factors due to the other remaining traits. We further conducted multivariable MR analysis, which estimates the effect of each exposure included in the estimation on the outcome conditional on the other exposure included in the model, that is, the direct effect of each exposure [64]. All the IVs used in the univariable MR analysis of short sleep duration, sleep chronotype and insomnia, a total of 140 IVs (Supplementary Table S3), were included in multivariable MR analysis. We found that after controlling for sleep chronotype and insomnia, short sleep duration still had a negative causal effect on lifespan (IVW: *β* = –0.59, 95%CI = –0.98 to –0.20, *P* = 0.003; MR-Egger: *β* = –0.79, 95%CI = –1.21 to –0.21, *P* = 0.006) (Table 2). The multivariable MR-Egger intercept term was close to zero, indicating no horizontal pleiotropy (*P* = 0.44). However, the heterogeneity test showed that there was heterogeneity across all the IVs (IVW, Q = 212.18, df = 137, *P* < 1 × 10^–4^), which might be because we included too many IVs in the MVMR analysis. No causal effect of sleep chronotype (IVW: *β* = 0.04, 95%CI = –0.08 to 0.17, *P* = 0.50; MR-Egger: *β* = 0.05, 95%CI = –0.08 to 0.18, *P* = 0.46) or insomnia (IVW: *β* = –0.002, 95%CI = –0.08 to 0.08, *P* = 0.96; MR-Egger: *β* = 0.001, 95%CI = –0.001 to 0.002, *P* = 0.44) on lifespan was observed when conditioning on other traits (Table 2).

**Table 2:** MVMR results of sleep behaviors conditioning of other sleep-related behaviors.

Method	Number of IVs	*β* (95%CI)	*P*
**Short sleep duration**			
IVW	140	–0.59 (–0.98 to –0.20)	0.003
MR-Egger		–0.79 (–1.21 to –0.21)	0.006
**Sleep chronotype**			
IVW	140	0.04 (–0.08 to 0.17)	0.50
MR-Egger		0.05 (–0.08 to 0.18)	0.46
**Insomnia**			
IVW	140	–0.002 (–0.08 to 0.08)	0.96
MR-Egger		0.001 (–0.001 to 0.002)	0.44

### Mediation analysis

We further attempted to analyze the causal effect and determine the causal pathways by which short sleep duration influences lifespan through mediation analysis. Briefly, mediation analysis is a two-step MR analysis that first estimates the causal effect between exposure and mediator and then estimates the causal effect between mediator and outcome. As disruption of circadian rhythms was reported to be associated with metabolic, cardiovascular, mental health and immunological functions [67], we selected CAD, AIS, depression, T2D, heart failure, atrial fibrillation and BMI as mediators and evaluated the relationships between these diseases and sleep behaviors/lifespan. We first assessed the causal relationship between short sleep duration and these mediators. The IVs used for mediation analysis are listed in Supplementary Table S4, and the results are summarized in Table 3 and Supplementary Table S5. We found that short sleep duration had a positive causal effect on CAD (IVW odds ratio (OR) = 2.35; 95% CI = 1.35 to 4.11; *P* = 0.003), T2D (IVW OR = 2.30; 95% CI = 1.16 to 4.58; *P* = 0.02) and depression (IVW OR = 2.69; 95% CI = 1.65 to 4.37; *P* = 6.94 × 10^–5^) (Table 3, Supplementary Figure S2). The MR-Egger, weighted median, weighted mode and MR.RAPS methods also provided consistent findings (Table 3, Supplementary Figure S2). The MR-Egger intercept analysis did not determine any pleiotropy effect (CAD: *P* = 0.99; T2D: *P* = 0.55; depression: *P* = 0.87). Cohran’s Q statistic indicated that there were heterogeneities between the IVs (CAD: Q = 41.1, df = 21, *P* = 0.005; T2D: Q = 38.5, df = 22, *P* = 0.02; depression: Q = 43.0, df = 19, *P* = 0.02). Leave-one-out analysis results showed that the causal effects were not driven by any one of the IVs (Supplementary Figure S2C, F and I). We also found that short sleep duration had a positive causal effect on BMI (IVW *β* = 0.24, 95%CI = 0.01 to 0.47, *P* = 0.04) and heart failure (IVW OR = 2.11, 95%CI = 1.09 to 4.07, *P* = 0.03) (Supplementary Figure S3 and Table S5). However, the causality might be driven by some IVs as the leave-one-out analysis results showed that the S.E. lines of some SNPs crossed zero (Supplementary Figure S3). Short sleep duration had no causal effect on atrial fibrillation (IVW OR = 1.56, 95%CI = 0.89 to 2.72, *P* = 0.12), AIS (IVW OR = 1.41, 95%CI = 0.70 to 2.84, *P* = 0.34), SZ (IVW OR = 1.21, 95%CI = 0.31 to 4.68, *P* = 0.79) and BD (IVW OR = 1.63, 95%CI = 0.58 to 4.34, *P* = 0.25) (Supplementary Figure S3 and Table S5).

**Table 3:** Mediation MR results of short sleep duration on CAD and T2D and further results of CAD and T2D on lifespan.

MR Method	Number of IVs	*F*	OR (95%CI)	*P*
**Short sleep duration on CAD**				
IVW	22	38	2.35 (1.35 to 4.11)	0.003
Weighted median			1.24 (0.68 to 2.26)	0.47
MR-Egger			2.38 (0.17 to 32.96)	0.53
Weighted mode			1.04 (0.46 to 2.33)	0.93
MR.RAPS			2.03 (1.18 to 3.49)	0.01
**Short sleep duration on T2D**				
IVW	23	38	2.30 (1.16 to 4.58)	0.02
Weighted median			1.79 (0.80 to 3.99)	0.16
MR-Egger			1.02 (0.06 to 16.02)	0.99
Weighted mode			0.59 (0.11 to 3.25)	0.55
MR.RAPS			2.26 (1.08 to 4.71)	0.03
**Short sleep duration on depression**				
IVW	20	38	2.69 (1.65 to 4.37)	6.94 × 10^–5^
Weighted median			2.08 (1.23 to 3.51)	0.006
MR-Egger			3.14 (0.51 to 19.44)	0.24
Weighted mode			1.64 (0.52 to 5.12)	0.41
MR.RAPS			2.96 (1.77 to 4.94)	3.36 × 10^–5^
**CAD on lifespan**			***β*** **(95%CI)**	
IVW	138	39	–0.20 (–0.22 to –0.18)	1.26 × 10^–80^
Weighted median			–0.18 (–0.21 to –0.15)	7.14 × 10^–39^
MR-Egger			–0.20 (–0.24 to –0.16)	2.45 × 10^–17^
Weighted mode			–0.15 (–0.21 to –0.09)	3.26 × 10^–6^
MR.RAPS			–0.21 (–0.23 to –0.19)	2.86 × 10^–88^
**T2D on lifespan**				
IVW	119	494	–0.02 (–0.03 to –0.009)	2.15 × 10^–4^
Weighted median			–0.02 (–0.04 to 0.004)	0.11
MR-Egger			–0.0005 (–0.02 to 0.02)	0.96
Weighted mode			–0.02 (–0.04 to 0.003)	0.1
MR.RAPS			–0.02 (–0.03 to –0.01)	1.08 × 10^–4^
**Depression on lifespan**				
IVW	43	38	−0.11 (−0.16 to −0.05)	1.67 × 10^–4^
Weighted median			−0.11 (−0.18 to −0.05)	7.13 × 10^–4^
MR-Egger			−0.09 (−0.44 to 0.26)	0.60
Weighted mode			−0.19 (−0.35 to −0.03)	0.02
MR.RAPS			−0.11 (−0.17 to −0.05)	4.25 × 10^–4^

As short sleep duration had positive causal effects on CAD, T2D and depression, we further conducted the second step of mediation analysis, which assessed the causal relationship between CAD, T2D, depression and lifespan. The results showed that both CAD (IVW *β* = –0.20, 95%CI = –0.22 to –0.18, *P* = 1.26 × 10^–80^), T2D (IVW *β* = –0.02, 95%CI = –0.03 to –0.009, *P* = 2.15 × 10^–4^) and depression (IVW *β* = –0.11, 95%CI = –0.16 to –0.05, *P* = 1.67 × 10^–4^) had negative causal effects on lifespan (Table 3 and Supplementary Figure S2). The final indirect causal effects from short sleep duration to lifespan mediated by CAD, depression and T2D were reflected by values of –0.17, –0.02 and –0.11 with proportions of 27.9%, 2.6% and 18.1%, respectively.

## Discussion

Conventional epidemiological studies have found increased rates of mortality among individuals with short sleep duration, and in the present study, using MR, we provided direct genetic evidence that short sleep duration is a causal risk factor for short lifespan, which is partially mediated by CAD and T2D. This result is largely robust according to the convergent results from different MR methods, and horizontal pleiotropy is thus unlikely to be an adequate explanation of our results.

Our results are also consistent with previous MR studies showing that short sleep duration is a causal risk factor for cardiovascular diseases and other poor physical and mental health [27, 68, 69]. Many previous studies have shown that insufficient sleep might impact future disease pathology in a number of ways, including increasing endothelial oxidative stress [70, 71], influencing immune and inflammatory responses [72, 73], altering gut microbiota composition and its metabolites [74]. Moreover, a recent study suggested that periods of poor sleep, even if followed by sleep recovery, have sustained negative consequences on human health [72]. Nevertheless, we showed that insomnia or sleep chronotype did not have any causal effects on lifespan, which seems to contrast with our first impression; however, it is likely that individuals with insomnia or “morning” people may have enough sleep time by taking naps during the daytime. These results again suggest that short sleep duration is harmful to our physical health, and we might be able to reduce the negative effects of insomnia by increasing sleep duration. Indeed, recent evidence has indicated that extended sleep duration could improve cardiovascular health among individuals with potential sleep deprivation [75, 76]. However, we also cannot exclude the possibility that the number of IVs of insomnia GWAS [33] is limited compared with duration short [31], hindering our ability to determine significant effects between insomnia and lifespan. Another explanation is the limited sample size of the current insomnia GWAS [33], which might reduce the statistical effectiveness. Further studies in larger samples are warranted to confirm the conclusions in the present study.

Although the results are intriguing, this study has certain limitations. For example, in the mediation analysis, although we found that CAD, T2D and depression could mediate the effects of short sleep duration on lifespan, there might be other risk factors. Secondly, we did not detect the causal effect of long sleep duration on lifespan, which is inconsistent with previous studies showing that sleep duration time exhibited a U- or J-shaped associations with higher rate of mortality [16, 17]. The most likely reason to explain the inconsistence is that the IVs used to infer the causality from long sleep duration to lifespan is too small to get a significant result. Future GWASs with larger sample size of long sleep duration might obtain more robust MR results on lifespan. Lastly, the definition of short sleep duration was self-reported rather than objectively measured, which might potentially bias the GWAS results to some extent, although there is a moderate to strong correlation between self-reported and objectively measured sleep duration according to a previous study [77]. With the popularity of smart wearable devices, we may be able to measure sleep conditions more accurately in the future.

In summary, we have provided robust evidence for the negative causal effects of genetically predicted short sleep duration on lifespan through MR analysis, which could minimize potential biases due to confounding factors and reverse causality in observational studies. We also showed that insomnia has no causal effects on lifespan. This collective evidence indicated that extension of sleep may benefit physical health for individuals with sleep loss, and further attention should be given to such public health issues.

### Supplementary information

SUPPLEMENTAL MATERIAL

## Data Availability

All the GWAS data and statistical software used in this study were publicly available, and all the generated results in this study were provided in the main text and supplemental data.
