# Supplementary material for: Shared genetic architecture and causal relationship between sleep behaviors and lifespan
Source: Transl Psychiatry. 2024 Feb 22;14:108. doi: 10.1038/s41398-024-02826-x (PMC10883970; doi:10.1038/s41398-024-02826-x)
Supplement: Supplementary file 1 — SUPPLEMENTAL MATERIAL [file 41398_2024_2826_MOESM1_ESM.pdf]

Figure S1. Two sample MR results of long sleep duration **(A, B, C)**, sleep chronotype **(D, E, F),** and insomnia on lifespan **(G, H, I)**. **(A), (D), (G)** Scatter plots of two sample MR. **(B), (E), (H)** Forest plots of the effect of each SNP. **(C), (F), (I)** Forest plots of leave-one-out results.

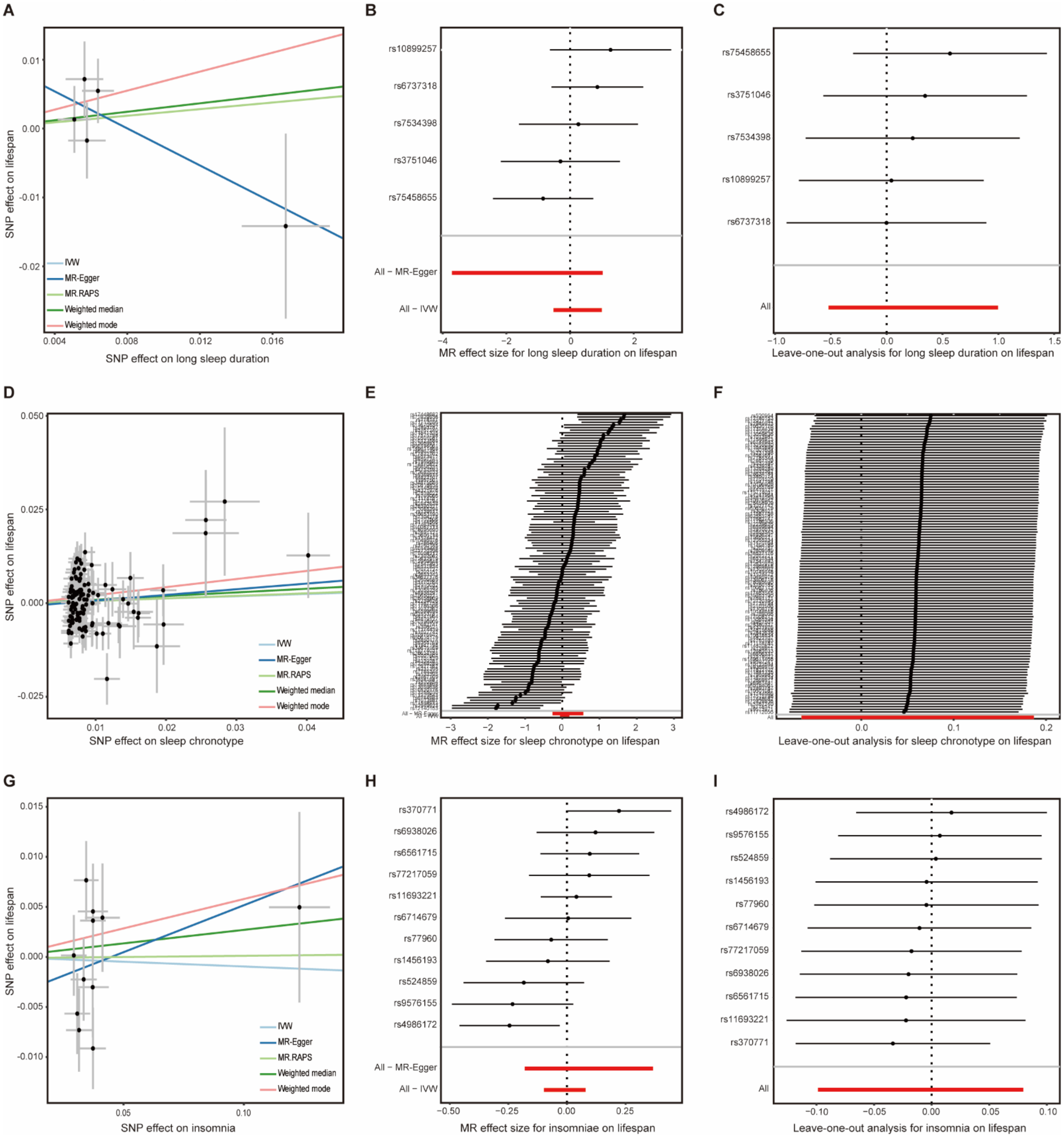

Figure S2. Two sample MR results of short sleep duration on CAD **(A, B, C),** T2D **(D, E, F)** and depression **(G, H, I),** and two sample MR results of CAD **(J, K, L)**, T2D **(M, N, O)** and depression **(P, Q, R)** on lifespan. **(A), (D), (G), (J), (M), (P)** Scatter plots of two sample MR. **(B), (E), (H), (K), (N), (Q)** Forest plots of the effect of each SNP. **(C), (F), (I), (L), (O), (R)** Forest plots of leave-one-out results.

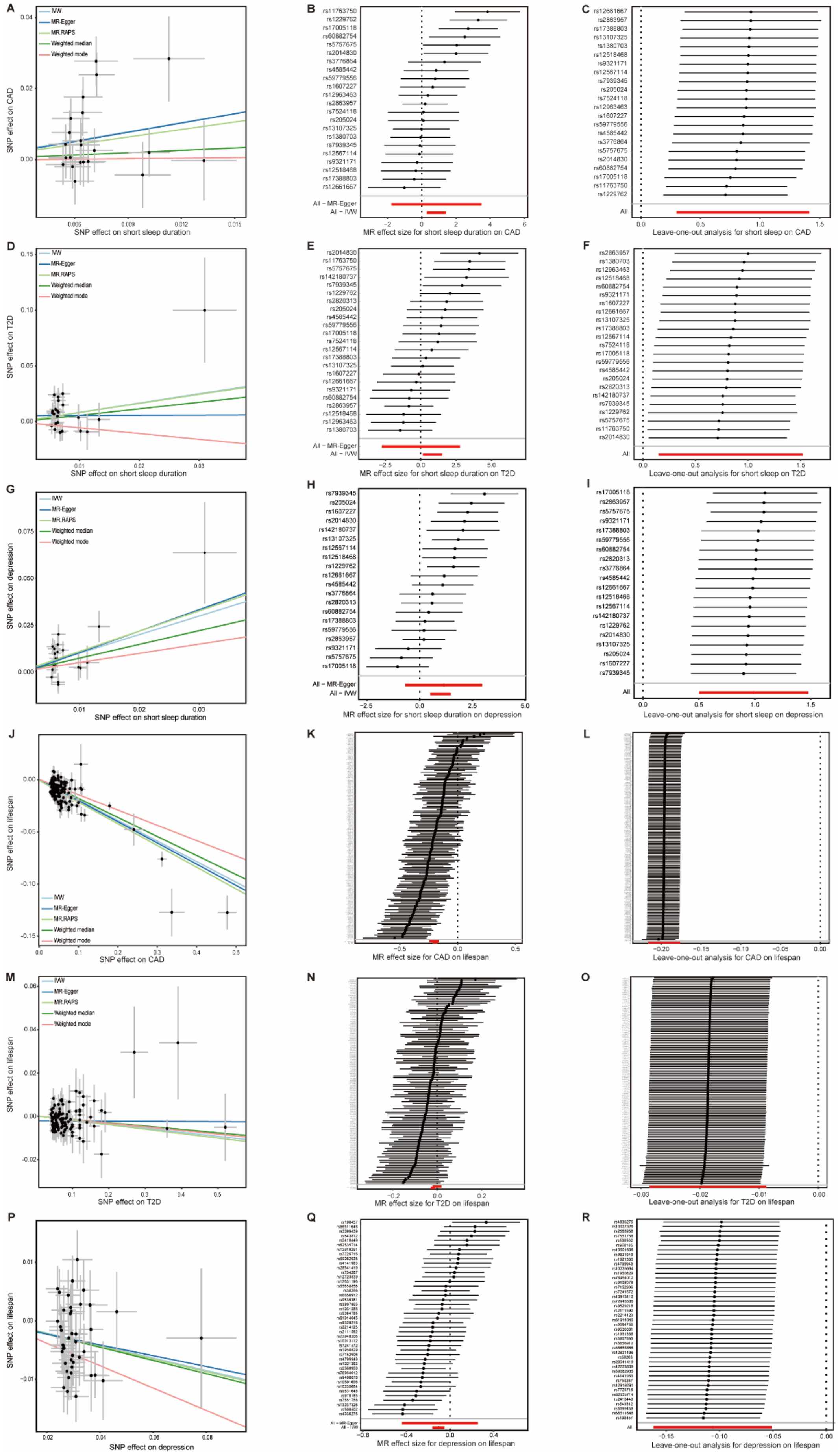

Figure S3. Two sample MR results of short sleep duration on BMI **(A, B, C)**, heart failure **(D, E, F)**, atrial fibrillation **(G, H, I)**, AIS **(J, K, L)**, schizophrenia **(M, N, O)**, bipolar disorder **(P, Q, R)**. **(A)**, **(D)**, **(G)**, **(J)**, **(M)**, **(P)** Scatter plots of two sample MR. **(B)**, **(E)**, **(H)**, **(K)**, **(N)**, **(Q)** Forest plots of the effect of each SNP. **(C)**, **(F)**, **(I)**, **(L)**, **(O)**, **(R)** Forest plots of leave-one-out results.

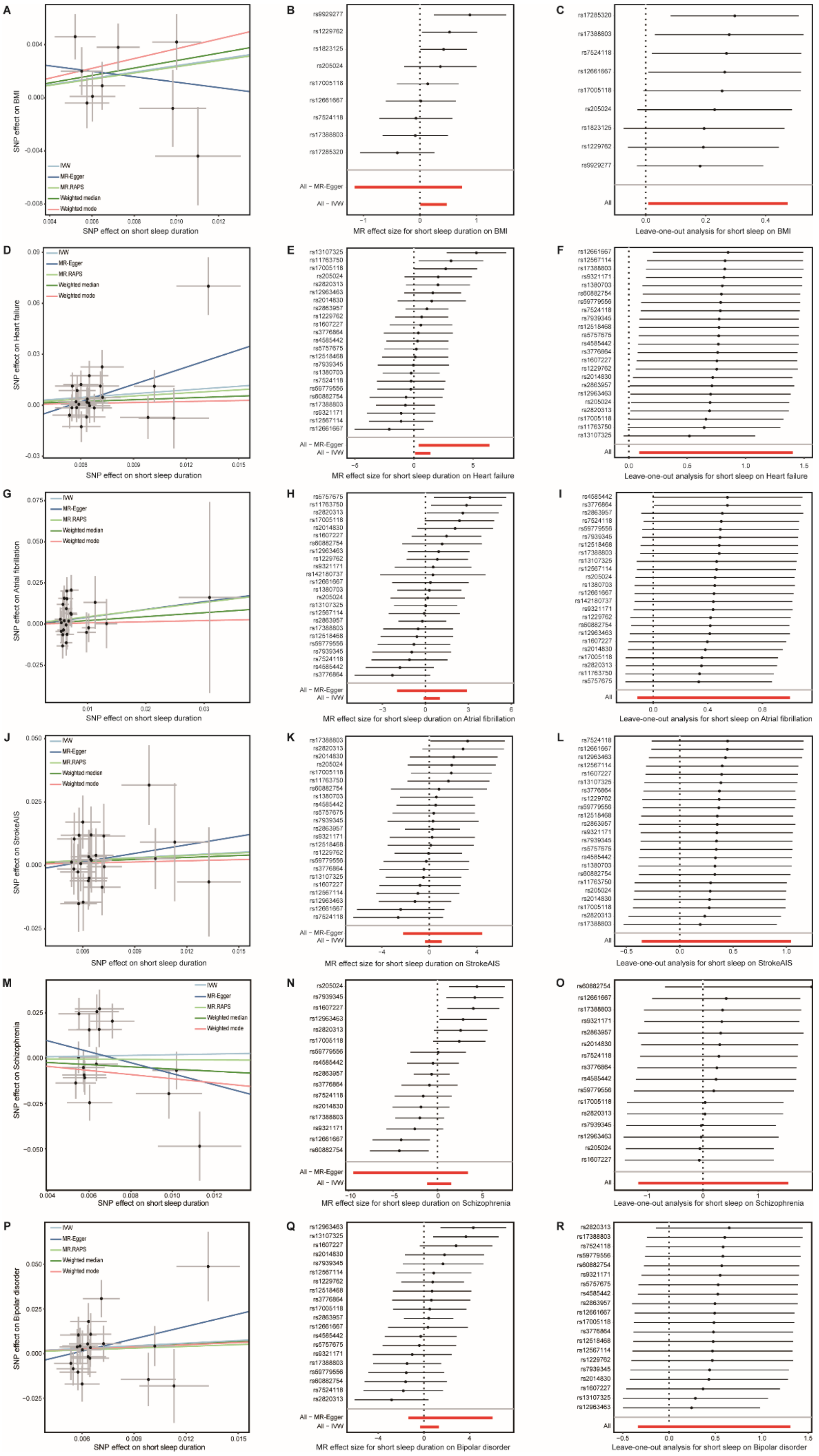

Table S1. Detailed information of GWASs used in this study

Phenotype	Case number	Control number	Total number	PMID	Download link
Short sleep duration	106,192	305,742	411,934	30846698	https://sleep.hugeamp.org/downloads.html
Long sleep duration	34,184	305,742	339,926	30846698	https://sleep.hugeamp.org/downloads.html
Sleep chronotype	252,287	150,908	403,195	30696823	https://sleep.hugeamp.org/downloads.html
Insomnia	109,548	277,440	386,988	35835914	https://ctg.cncr.nl/software/summary_statistics/
Lifespan	-	-	1,012,240	30642433	http://dx.doi.org/10.7488/ds/2463
Coronary artery disease (CAD)	122,733	424,528	547,261	29212778	http://www.cardiogramplusc4d.org/data-downloads/
Any ischemic stroke (AIS)	34,217	406,111	440,328	29531354	http://www.megastroke.org/download.html
Type 2 diabetes (T2D)	74,124	824,006	898,130	30297969	http://diagram-consortium.org/downloads.html
Heart failure	47,309	930,014	977,323	31919418	https://cvd.hugeamp.org/downloads.html
Atrial fibrillation	65,446 (55,114 from European)	522,744 (482,295 from European)	588,190	29892015	https://cvd.hugeamp.org/downloads.html
Body mass index (BMI)	-	-	681,275	30124842	https://cnsgenomics.com/content/data
Depression	170,756	329,443	500,199	30718901	https://pgc.unc.edu/for-researchers/download-results/
Bipolar disorder (BD)	41,917	371,549	413,466	34002096	https://pgc.unc.edu/for-researchers/download-results/
Schizophrenia (SZ)	53,386	77,258	130,644	35396580	https://pgc.unc.edu/for-researchers/download-results/

Table S2. Genetical instrumental variables used in the univariable MR analysis

NO	SNP	Effect Allele	Other Allele	Exposure					Outcome				
				Beta	SE	P	Exposure	Sample Size	Beta	SE	P	Outcome	Sample Size
1	rs11763750	G	A	0.0072	0.0012	5.10E−09	Duration Short	411934	−0.0146	0.0049	0.0030	Lifespan	638110
2	rs1229762	C	T	−0.0072	0.0010	1.00E−12	Duration Short	411934	0.0128	0.0041	0.0018	Lifespan	638116
3	rs12518468	T	C	−0.0059	0.0010	8.50E−09	Duration Short	411934	0.0000	0.0041	0.9962	Lifespan	637314
4	rs12567114	G	A	0.0063	0.0011	4.10E−09	Duration Short	411934	−0.0030	0.0044	0.4946	Lifespan	640145
5	rs12661667	C	T	−0.0060	0.0011	2.80E−08	Duration Short	411934	0.0003	0.0044	0.9444	Lifespan	640181
6	rs13107325	C	T	−0.0133	0.0018	2.50E−13	Duration Short	411934	0.0073	0.0074	0.3267	Lifespan	640179
7	rs1380703	A	G	−0.0068	0.0010	1.60E−11	Duration Short	411934	−0.0046	0.0041	0.2529	Lifespan	640185
8	rs142180737	T	C	−0.0309	0.0053	4.40E−09	Duration Short	411934	0.0198	0.0240	0.4080	Lifespan	355876
9	rs1607227	G	T	0.0064	0.0011	1.50E−09	Duration Short	411934	−0.0068	0.0043	0.1143	Lifespan	637314
10	rs17005118	G	A	−0.0065	0.0011	2.50E−09	Duration Short	411934	0.0133	0.0044	0.0025	Lifespan	638114
11	rs17388803	A	C	−0.0098	0.0016	6.50E−10	Duration Short	411934	−0.0010	0.0064	0.8733	Lifespan	635243
12	rs2014830	C	T	0.0058	0.0011	2.70E−08	Duration Short	411934	−0.0076	0.0042	0.0697	Lifespan	640032
13	rs205024	C	T	0.0055	0.0010	2.70E−08	Duration Short	411934	−0.0017	0.0040	0.6694	Lifespan	640176
15	rs2820313	A	G	−0.0060	0.0010	2.30E−09	Duration Short	411934	0.0036	0.0041	0.3719	Lifespan	640138
16	rs2863957	C	A	0.0102	0.0012	2.60E−18	Duration Short	411934	−0.0049	0.0047	0.3008	Lifespan	623282
17	rs3776864	A	C	0.0057	0.0010	1.70E−08	Duration Short	411934	−0.0016	0.0041	0.6971	Lifespan	637314
18	rs4585442	A	G	−0.0063	0.0010	8.10E−10	Duration Short	411934	−0.0017	0.0042	0.6771	Lifespan	637314
19	rs5757675	G	T	0.0065	0.0011	2.70E−09	Duration Short	411934	−0.0125	0.0044	0.0048	Lifespan	637314
20	rs59779556	T	G	0.0055	0.0010	2.00E−08	Duration Short	411934	0.0048	0.0039	0.2192	Lifespan	640161
21	rs60882754	A	T	0.0113	0.0020	1.80E−08	Duration Short	411934	−0.0015	0.0081	0.8492	Lifespan	639154
22	rs7524118	T	C	−0.0058	0.0011	4.90E−08	Duration Short	411934	0.0043	0.0042	0.3074	Lifespan	640187
23	rs9321171	C	T	0.0054	0.0010	4.20E−08	Duration Short	411934	−0.0040	0.0039	0.3017	Lifespan	638072
1	rs10899257	G	A	−0.0056	0.0010	4.60E−08	Duration Long	339926	−0.0072	0.0055	0.1900	Lifespan	640179
2	rs3751046	A	G	−0.0058	0.0010	2.00E−08	Duration Long	339926	0.0017	0.0055	0.7523	Lifespan	621213
3	rs6737318	A	G	−0.0064	0.0009	3.40E−13	Duration Long	339926	−0.0055	0.0047	0.2422	Lifespan	637314
4	rs7534398	T	A	−0.0051	0.0009	2.10E−08	Duration Long	339926	−0.0013	0.0048	0.7839	Lifespan	639040
5	rs75458655	C	T	−0.0167	0.0024	5.40E−12	Duration Long	339926	0.0142	0.0134	0.2915	Lifespan	631831
1	rs10067113	C	T	−0.0066	0.0011	2.50E−09	Sleep Chronotype	403195	−0.0019	0.0040	0.6250	Lifespan	640187
2	rs10123584	G	A	0.0063	0.0012	4.00E−08	Sleep Chronotype	403195	0.0066	0.0042	0.1221	Lifespan	638114
3	rs1013987	T	C	−0.0063	0.0011	4.50E−09	Sleep Chronotype	403195	0.0080	0.0039	0.0435	Lifespan	626114
4	rs10148448	G	A	−0.0066	0.0012	5.00E−08	Sleep Chronotype	403195	−0.0031	0.0045	0.4995	Lifespan	639993
5	rs10149448	A	G	0.0064	0.0011	5.60E−09	Sleep Chronotype	403195	0.0015	0.0040	0.7132	Lifespan	638106

6	rs10196909	C	A	0.0075	0.0011	6.20E−13	Sleep Chronotype	403195	−0.0026	0.0039	0.4947	Lifespan	638114
7	rs1027742	A	G	0.0072	0.0012	3.60E−09	Sleep Chronotype	403195	−0.0033	0.0044	0.4528	Lifespan	640154
8	rs10495976	A	T	−0.0066	0.0011	8.00E−10	Sleep Chronotype	403195	−0.0017	0.0040	0.6695	Lifespan	637314
9	rs10501087	T	C	−0.0081	0.0013	5.40E−10	Sleep Chronotype	403195	−0.0089	0.0048	0.0624	Lifespan	640189
10	rs10520176	T	C	0.0078	0.0011	2.00E−13	Sleep Chronotype	403195	−0.0072	0.0039	0.0623	Lifespan	640173
11	rs10818834	T	C	0.0076	0.0012	4.20E−10	Sleep Chronotype	403195	0.0035	0.0043	0.4181	Lifespan	640180
12	rs10976942	C	A	−0.0116	0.0019	1.00E−09	Sleep Chronotype	403195	0.0053	0.0069	0.4408	Lifespan	638112
13	rs11032362	G	A	−0.0158	0.0018	7.30E−18	Sleep Chronotype	403195	0.0039	0.0067	0.5607	Lifespan	638099
14	rs11174781	T	C	0.0111	0.0016	3.90E−12	Sleep Chronotype	403195	0.0049	0.0059	0.4097	Lifespan	638112
15	rs11229543	G	A	0.0079	0.0012	1.10E−10	Sleep Chronotype	403195	−0.0089	0.0045	0.0484	Lifespan	640188
16	rs1144566	T	C	0.0399	0.0031	2.80E−38	Sleep Chronotype	403195	0.0127	0.0114	0.2634	Lifespan	638062
17	rs114870822	G	A	−0.0254	0.0047	3.40E−08	Sleep Chronotype	403195	−0.0187	0.0168	0.2676	Lifespan	636736
18	rs115774037	T	C	−0.0184	0.0033	3.50E−08	Sleep Chronotype	403195	0.0115	0.0124	0.3543	Lifespan	637314
19	rs11587758	G	A	−0.0081	0.0011	3.80E−14	Sleep Chronotype	403195	0.0018	0.0039	0.6540	Lifespan	638105
20	rs11588913	G	A	0.0059	0.0011	2.50E−08	Sleep Chronotype	403195	−0.0079	0.0039	0.0433	Lifespan	640181
21	rs11645898	T	C	0.0084	0.0014	1.80E−09	Sleep Chronotype	403195	−0.0075	0.0052	0.1481	Lifespan	640138
22	rs11670534	C	T	0.0077	0.0014	4.70E−08	Sleep Chronotype	403195	0.0106	0.0052	0.0434	Lifespan	638074
23	rs11712056	T	C	0.0075	0.0011	3.70E−12	Sleep Chronotype	403195	0.0115	0.0039	0.0031	Lifespan	640142
24	rs11786306	G	C	−0.0068	0.0011	7.00E−10	Sleep Chronotype	403195	0.0016	0.0041	0.6857	Lifespan	638063
25	rs11841335	G	A	0.0066	0.0012	4.00E−08	Sleep Chronotype	403195	0.0082	0.0044	0.0646	Lifespan	638118
26	rs12055234	G	A	−0.0064	0.0011	1.50E−08	Sleep Chronotype	403195	0.0009	0.0041	0.8260	Lifespan	640147
27	rs12140153	G	T	0.0114	0.0018	4.80E−10	Sleep Chronotype	403195	−0.0202	0.0069	0.0032	Lifespan	636048
28	rs12669911	A	C	−0.0069	0.0011	1.80E−10	Sleep Chronotype	403195	−0.0070	0.0040	0.0794	Lifespan	633039
29	rs12682033	T	C	0.0082	0.0012	6.10E−11	Sleep Chronotype	403195	0.0048	0.0046	0.2892	Lifespan	626139
30	rs12927162	A	G	0.0108	0.0012	2.00E−20	Sleep Chronotype	403195	−0.0081	0.0044	0.0670	Lifespan	640183
31	rs12969848	C	T	−0.0085	0.0011	2.60E−15	Sleep Chronotype	403195	−0.0009	0.0039	0.8101	Lifespan	638094
32	rs13059636	A	G	−0.0074	0.0011	3.50E−12	Sleep Chronotype	403195	0.0068	0.0039	0.0796	Lifespan	640165
33	rs13065394	G	T	0.0070	0.0012	2.20E−09	Sleep Chronotype	403195	0.0029	0.0043	0.5030	Lifespan	638090
34	rs13255030	A	G	0.0061	0.0011	1.50E−08	Sleep Chronotype	403195	−0.0061	0.0039	0.1216	Lifespan	640175
35	rs13269289	G	A	−0.0065	0.0011	5.50E−09	Sleep Chronotype	403195	0.0045	0.0042	0.2874	Lifespan	627189
36	rs139911	C	T	0.0088	0.0011	2.20E−16	Sleep Chronotype	403195	0.0033	0.0039	0.3976	Lifespan	638067
37	rs1470764	G	A	0.0067	0.0011	5.90E−10	Sleep Chronotype	403195	−0.0006	0.0040	0.8704	Lifespan	638097
38	rs1494185	G	A	0.0067	0.0012	1.10E−08	Sleep Chronotype	403195	−0.0008	0.0043	0.8492	Lifespan	638116
39	rs149611468	T	C	0.0281	0.0049	1.30E−08	Sleep Chronotype	403195	0.0271	0.0198	0.1705	Lifespan	403799
40	rs1524472	A	G	0.0059	0.0011	2.10E−08	Sleep Chronotype	403195	0.0017	0.0039	0.6692	Lifespan	640153
41	rs17374439	C	T	−0.0130	0.0013	1.20E−22	Sleep Chronotype	403195	0.0058	0.0048	0.2290	Lifespan	638108

42	rs17448682	C	T	−0.0071	0.0012	8.30E−09	Sleep Chronotype	403195	−0.0119	0.0046	0.0095	Lifespan	637314
43	rs17463545	C	T	−0.0121	0.0019	4.40E−10	Sleep Chronotype	403195	−0.0037	0.0073	0.6109	Lifespan	635245
44	rs17575798	G	A	0.0092	0.0013	7.80E−12	Sleep Chronotype	403195	0.0102	0.0049	0.0374	Lifespan	638107
45	rs17682747	G	A	−0.0072	0.0013	8.70E−09	Sleep Chronotype	403195	0.0026	0.0046	0.5638	Lifespan	640135
46	rs17822102	A	G	−0.0070	0.0011	1.30E−10	Sleep Chronotype	403195	−0.0066	0.0041	0.1043	Lifespan	638087
47	rs1947198	C	T	−0.0093	0.0016	3.20E−09	Sleep Chronotype	403195	0.0054	0.0059	0.3651	Lifespan	640161
48	rs202157	C	T	0.0093	0.0012	1.30E−15	Sleep Chronotype	403195	0.0000	0.0042	0.9963	Lifespan	638108
49	rs2072727	T	C	0.0065	0.0011	5.60E−10	Sleep Chronotype	403195	0.0000	0.0039	0.9959	Lifespan	629371
50	rs2102506	G	A	0.0069	0.0011	8.30E−10	Sleep Chronotype	403195	0.0022	0.0040	0.5841	Lifespan	637312
51	rs2239626	T	C	−0.0079	0.0011	5.00E−12	Sleep Chronotype	403195	0.0014	0.0042	0.7468	Lifespan	624051
52	rs231398	G	A	0.0083	0.0014	5.80E−09	Sleep Chronotype	403195	−0.0064	0.0053	0.2268	Lifespan	637314
53	rs2467109	T	A	−0.0065	0.0012	3.90E−08	Sleep Chronotype	403195	0.0052	0.0043	0.2273	Lifespan	638083
54	rs2518022	T	C	0.0147	0.0019	7.60E−15	Sleep Chronotype	403195	0.0067	0.0069	0.3310	Lifespan	638114
55	rs2609589	T	C	−0.0084	0.0015	3.60E−08	Sleep Chronotype	403195	0.0004	0.0055	0.9484	Lifespan	640180
56	rs2653343	T	A	0.0144	0.0013	5.20E−29	Sleep Chronotype	403195	−0.0001	0.0047	0.9808	Lifespan	638106
57	rs28380327	A	T	0.0068	0.0011	7.80E−10	Sleep Chronotype	403195	0.0028	0.0040	0.4834	Lifespan	638085
58	rs2842638	T	G	0.0072	0.0011	1.90E−11	Sleep Chronotype	403195	0.0030	0.0040	0.4528	Lifespan	637314
59	rs28458909	C	T	0.0158	0.0016	4.70E−23	Sleep Chronotype	403195	−0.0027	0.0064	0.6732	Lifespan	535216
60	rs28634184	C	T	0.0070	0.0012	1.30E−08	Sleep Chronotype	403195	−0.0044	0.0045	0.3277	Lifespan	623282
61	rs2893787	G	A	0.0068	0.0012	3.50E−08	Sleep Chronotype	403195	0.0090	0.0044	0.0413	Lifespan	640178
62	rs2910032	C	T	−0.0079	0.0011	1.10E−13	Sleep Chronotype	403195	0.0008	0.0039	0.8265	Lifespan	638117
63	rs2949923	A	G	−0.0062	0.0011	4.20E−09	Sleep Chronotype	403195	0.0108	0.0039	0.0056	Lifespan	637314
64	rs308521	T	C	0.0065	0.0011	1.50E−09	Sleep Chronotype	403195	−0.0026	0.0040	0.5168	Lifespan	633206
65	rs34581681	G	A	0.0081	0.0014	4.20E−08	Sleep Chronotype	403195	−0.0066	0.0053	0.2136	Lifespan	640130
66	rs34619169	G	A	−0.0064	0.0011	1.50E−08	Sleep Chronotype	403195	0.0038	0.0042	0.3678	Lifespan	628619
67	rs34627176	G	A	−0.0070	0.0013	3.60E−08	Sleep Chronotype	403195	0.0002	0.0048	0.9650	Lifespan	626686
68	rs34875688	T	A	0.0075	0.0013	1.10E−09	Sleep Chronotype	403195	0.0035	0.0046	0.4455	Lifespan	640160
69	rs35653190	C	T	0.0071	0.0013	2.00E−08	Sleep Chronotype	403195	0.0025	0.0046	0.5870	Lifespan	628148
70	rs3760185	C	T	0.0087	0.0012	2.10E−12	Sleep Chronotype	403195	0.0021	0.0046	0.6506	Lifespan	635243
71	rs3767240	T	C	−0.0073	0.0011	7.30E−12	Sleep Chronotype	403195	−0.0093	0.0040	0.0192	Lifespan	633249
72	rs3850174	T	A	0.0075	0.0012	1.40E−09	Sleep Chronotype	403195	−0.0041	0.0045	0.3676	Lifespan	621211
73	rs4241964	T	G	−0.0076	0.0011	7.20E−13	Sleep Chronotype	403195	0.0025	0.0039	0.5179	Lifespan	637314
74	rs4321976	T	C	0.0077	0.0013	8.70E−10	Sleep Chronotype	403195	0.0035	0.0047	0.4491	Lifespan	633092
75	rs4339281	A	G	0.0092	0.0016	5.30E−09	Sleep Chronotype	403195	−0.0059	0.0058	0.3076	Lifespan	640177
76	rs4419127	A	G	0.0080	0.0011	5.70E−13	Sleep Chronotype	403195	−0.0027	0.0041	0.5007	Lifespan	638083
77	rs4752593	G	C	0.0062	0.0011	9.80E−09	Sleep Chronotype	403195	0.0037	0.0040	0.3543	Lifespan	638094

78	rs4822107	G	A	−0.0065	0.0011	8.70E−10	Sleep Chronotype	403195	−0.0033	0.0039	0.3941	Lifespan	637314
79	rs4936291	A	G	−0.0065	0.0011	9.40E−09	Sleep Chronotype	403195	0.0009	0.0041	0.8213	Lifespan	635243
80	rs520954	A	G	−0.0099	0.0011	1.60E−18	Sleep Chronotype	403195	0.0081	0.0041	0.0471	Lifespan	638104
81	rs532395	C	T	−0.0063	0.0012	2.30E−08	Sleep Chronotype	403195	0.0050	0.0042	0.2297	Lifespan	640169
82	rs60194061	G	A	−0.0076	0.0012	8.60E−11	Sleep Chronotype	403195	−0.0077	0.0044	0.0791	Lifespan	635243
83	rs60616179	A	G	0.0132	0.0023	9.20E−09	Sleep Chronotype	403195	−0.0062	0.0085	0.4686	Lifespan	638219
84	rs62553781	C	T	0.0194	0.0029	3.00E−11	Sleep Chronotype	403195	−0.0057	0.0107	0.5962	Lifespan	629711
85	rs6537834	T	C	−0.0059	0.0011	3.50E−08	Sleep Chronotype	403195	−0.0002	0.0040	0.9586	Lifespan	640173
86	rs6599694	G	T	0.0062	0.0011	2.10E−08	Sleep Chronotype	403195	−0.0017	0.0041	0.6745	Lifespan	640174
87	rs6656331	C	T	0.0064	0.0011	1.50E−09	Sleep Chronotype	403195	0.0045	0.0039	0.2454	Lifespan	640177
88	rs6744983	G	T	−0.0059	0.0011	4.90E−08	Sleep Chronotype	403195	0.0074	0.0040	0.0633	Lifespan	638110
89	rs6967481	C	T	−0.0070	0.0011	1.90E−11	Sleep Chronotype	403195	−0.0066	0.0039	0.0904	Lifespan	633236
90	rs7001604	T	C	−0.0075	0.0011	7.30E−12	Sleep Chronotype	403195	0.0047	0.0040	0.2408	Lifespan	637314
91	rs72632979	A	G	0.0081	0.0014	4.00E−09	Sleep Chronotype	403195	0.0010	0.0053	0.8527	Lifespan	637314
92	rs72720396	A	G	−0.0101	0.0013	5.30E−16	Sleep Chronotype	403195	−0.0021	0.0046	0.6422	Lifespan	635243
93	rs72829936	G	A	−0.0083	0.0014	5.50E−09	Sleep Chronotype	403195	−0.0136	0.0053	0.0097	Lifespan	623282
94	rs7302062	T	C	0.0074	0.0011	5.20E−12	Sleep Chronotype	403195	0.0011	0.0039	0.7776	Lifespan	638098
95	rs7304278	A	G	−0.0077	0.0012	9.60E−11	Sleep Chronotype	403195	−0.0025	0.0043	0.5602	Lifespan	637314
96	rs73606718	G	A	0.0094	0.0016	5.70E−09	Sleep Chronotype	403195	0.0027	0.0060	0.6473	Lifespan	637314
97	rs7547493	A	G	−0.0136	0.0014	2.50E−23	Sleep Chronotype	403195	−0.0011	0.0050	0.8341	Lifespan	640161
98	rs75650221	C	T	−0.0152	0.0028	3.30E−08	Sleep Chronotype	403195	0.0023	0.0100	0.8170	Lifespan	640178
99	rs769066	T	C	−0.0077	0.0014	1.90E−08	Sleep Chronotype	403195	−0.0034	0.0050	0.4944	Lifespan	635245
100	rs7691121	C	G	0.0080	0.0012	1.00E−10	Sleep Chronotype	403195	0.0023	0.0046	0.6096	Lifespan	640148
101	rs77008212	A	G	0.0193	0.0019	1.10E−24	Sleep Chronotype	403195	0.0034	0.0069	0.6188	Lifespan	631492
102	rs7701529	A	T	−0.0070	0.0012	1.70E−08	Sleep Chronotype	403195	0.0044	0.0046	0.3306	Lifespan	635243
103	rs7735794	G	A	−0.0074	0.0013	4.50E−08	Sleep Chronotype	403195	−0.0032	0.0052	0.5399	Lifespan	576191
104	rs778147	C	A	0.0070	0.0011	4.60E−10	Sleep Chronotype	403195	0.0109	0.0040	0.0067	Lifespan	637314
105	rs7785344	T	C	−0.0059	0.0011	2.60E−08	Sleep Chronotype	403195	0.0047	0.0039	0.2286	Lifespan	638108
106	rs78095690	T	C	−0.0065	0.0011	1.10E−09	Sleep Chronotype	403195	−0.0019	0.0039	0.6281	Lifespan	637314
107	rs786406	A	G	−0.0077	0.0012	1.00E−11	Sleep Chronotype	403195	−0.0020	0.0042	0.6415	Lifespan	635245
108	rs7959983	T	C	−0.0075	0.0011	4.70E−12	Sleep Chronotype	403195	−0.0060	0.0039	0.1259	Lifespan	640164
109	rs9365769	A	G	0.0062	0.0011	7.30E−09	Sleep Chronotype	403195	−0.0035	0.0039	0.3684	Lifespan	640187
110	rs9369915	G	A	−0.0087	0.0012	3.00E−14	Sleep Chronotype	403195	−0.0051	0.0042	0.2294	Lifespan	635243
111	rs9573971	A	G	0.0254	0.0029	4.90E−18	Sleep Chronotype	403195	0.0222	0.0107	0.0378	Lifespan	638116
112	rs957501	T	A	0.0062	0.0011	3.00E−08	Sleep Chronotype	403195	0.0030	0.0041	0.4676	Lifespan	640147
113	rs9597241	A	C	0.0076	0.0014	2.70E−08	Sleep Chronotype	403195	0.0066	0.0049	0.1842	Lifespan	638921

1	rs11693221	T	C	0.1231	0.0126	1.86E−22	Insomnia	377767	0.0050	0.0095	0.6019	Lifespan	637314
2	rs1456193	T	C	−0.0372	0.0067	2.40E−08	Insomnia	384257	0.0030	0.0050	0.5450	Lifespan	640187
3	rs370771	G	T	−0.0345	0.0051	1.54E−11	Insomnia	385767	−0.0077	0.0039	0.0503	Lifespan	635243
4	rs4986172	T	C	0.0373	0.0054	4.52E−12	Insomnia	386988	−0.0091	0.0041	0.0246	Lifespan	638112
5	rs524859	A	G	−0.0308	0.0053	6.83E−09	Insomnia	386988	0.0057	0.0040	0.1605	Lifespan	640179
6	rs6561715	T	A	0.0373	0.0053	3.67E−12	Insomnia	381990	0.0036	0.0040	0.3647	Lifespan	636802
7	rs6714679	C	T	−0.0293	0.0053	3.35E−08	Insomnia	381594	−0.0001	0.0040	0.9705	Lifespan	635243
8	rs6938026	G	A	0.0373	0.0062	1.70E−09	Insomnia	385631	0.0045	0.0048	0.3409	Lifespan	631430
9	rs77217059	A	G	−0.0412	0.0072	1.27E−08	Insomnia	379773	−0.0039	0.0054	0.4678	Lifespan	635243
10	rs77960	A	G	0.0334	0.0054	8.82E−10	Insomnia	383024	−0.0022	0.0041	0.5859	Lifespan	640171
11	rs9576155	A	G	0.0315	0.0054	8.00E−09	Insomnia	383478	−0.0073	0.0042	0.0783	Lifespan	633977

Table S3. Genetical instrumental variables used in the MVMR analysis

			Duration Short			Chronotype			Insomnia		
SNP	A1	A2	Beta	SE	*P*	Beta	SE	*P*	Beta	SE	*P*
rs10067113	C	T	0.0004	0.0010	6.40E−01	−0.0066	0.0011	2.50E−09	0.0050	0.0053	3.20E−01
rs10123584	G	A	−0.0005	0.0011	6.30E−01	0.0063	0.0012	4.00E−08	0.0020	0.0056	6.87E−01
rs1013987	T	C	−0.0013	0.0010	1.90E−01	−0.0063	0.0011	4.50E−09	0.0030	0.0052	5.10E−01
rs10148448	G	A	0.0020	0.0011	7.60E−02	−0.0066	0.0012	5.00E−08	−0.0030	0.0059	6.36E−01
rs10149448	A	G	−0.0016	0.0010	9.60E−02	0.0064	0.0011	5.60E−09	0.0030	0.0052	5.70E−01
rs10196909	C	A	0.0018	0.0010	6.60E−02	0.0075	0.0011	6.20E−13	−0.0061	0.0051	2.27E−01
rs1027742	A	G	0.0019	0.0011	8.10E−02	0.0072	0.0012	3.60E−09	0.0117	0.0058	4.55E−02
rs10495976	A	T	0.0002	0.0010	8.00E−01	−0.0066	0.0011	8.00E−10	0.0056	0.0053	2.89E−01
rs10501087	T	C	−0.0006	0.0012	6.10E−01	−0.0081	0.0013	5.40E−10	−0.0149	0.0063	1.60E−02
rs10520176	T	C	0.0022	0.0010	2.10E−02	0.0078	0.0011	2.00E−13	−0.0118	0.0051	2.09E−02
rs10818834	T	C	−0.0018	0.0011	1.20E−01	0.0076	0.0012	4.20E−10	−0.0090	0.0058	1.20E−01
rs10976942	C	A	0.0036	0.0017	4.00E−02	−0.0116	0.0019	1.00E−09	0.0033	0.0092	7.22E−01
rs11032362	G	A	0.0005	0.0017	7.30E−01	−0.0158	0.0018	7.30E−18	−0.0040	0.0088	6.77E−01
rs11174781	T	C	0.0015	0.0015	3.00E−01	0.0111	0.0016	3.90E−12	0.0000	0.0078	9.90E−01
rs11229543	G	A	0.0009	0.0011	4.60E−01	0.0079	0.0012	1.10E−10	0.0095	0.0060	1.11E−01
rs1144566	T	C	0.0012	0.0028	6.70E−01	0.0399	0.0031	2.80E−38	0.0159	0.0149	2.71E−01
rs114870822	G	A	−0.0036	0.0043	4.00E−01	−0.0254	0.0047	3.40E−08	−0.0227	0.0225	3.12E−01
rs115774037	T	C	−0.0039	0.0030	1.90E−01	−0.0184	0.0033	3.50E−08	0.0004	0.0160	9.78E−01
rs11587758	G	A	0.0005	0.0010	6.30E−01	−0.0081	0.0011	3.80E−14	0.0093	0.0052	7.25E−02
rs11588913	G	A	0.0010	0.0010	3.10E−01	0.0059	0.0011	2.50E−08	0.0048	0.0052	3.57E−01
rs11645898	T	C	−0.0001	0.0013	8.90E−01	0.0084	0.0014	1.80E−09	−0.0060	0.0068	3.99E−01
rs11670534	C	T	−0.0007	0.0013	6.00E−01	0.0077	0.0014	4.70E−08	0.0151	0.0069	2.88E−02
rs11693221	C	T	−0.0046	0.0023	5.00E−02	0.0107	0.0026	1.60E−05	−0.1231	0.0126	1.86E−22
rs11712056	T	C	−0.0021	0.0010	2.70E−02	0.0075	0.0011	3.70E−12	−0.0129	0.0051	1.06E−02
rs11763750	G	A	0.0072	0.0012	5.10E−09	0.0011	0.0014	4.20E−01	0.0233	0.0066	3.80E−04
rs11786306	G	C	0.0014	0.0010	1.80E−01	−0.0068	0.0011	7.00E−10	−0.0030	0.0054	5.68E−01
rs11841335	G	A	0.0007	0.0011	5.60E−01	0.0066	0.0012	4.00E−08	−0.0080	0.0059	1.75E−01
rs12055234	G	A	−0.0026	0.0010	8.70E−03	−0.0064	0.0011	1.50E−08	−0.0305	0.0054	1.71E−08
rs12140153	G	T	0.0023	0.0017	1.80E−01	0.0114	0.0018	4.80E−10	0.0151	0.0092	1.01E−01
rs1229762	C	T	−0.0072	0.0010	1.00E−12	−0.0041	0.0011	5.00E−04	−0.0246	0.0054	5.24E−06
rs12518468	T	C	−0.0059	0.0010	8.50E−09	0.0033	0.0011	2.20E−03	−0.0060	0.0054	2.46E−01
rs12567114	G	A	0.0063	0.0011	4.10E−09	−0.0031	0.0012	1.20E−02	0.0031	0.0057	5.92E−01
rs12661667	C	T	−0.0060	0.0011	2.80E−08	0.0050	0.0012	2.20E−05	−0.0100	0.0058	7.48E−02
rs12669911	A	C	−0.0016	0.0010	1.00E−01	−0.0069	0.0011	1.80E−10	0.0040	0.0053	4.00E−01
rs12682033	T	C	−0.0029	0.0011	9.00E−03	0.0082	0.0012	6.10E−11	−0.0173	0.0060	4.00E−03
rs12927162	A	G	−0.0027	0.0011	1.10E−02	0.0108	0.0012	2.00E−20	−0.0070	0.0057	2.08E−01
rs12969848	C	T	0.0003	0.0010	7.60E−01	−0.0085	0.0011	2.60E−15	−0.0080	0.0051	1.18E−01
rs13059636	A	G	0.0007	0.0010	4.50E−01	−0.0074	0.0011	3.50E−12	−0.0060	0.0052	2.70E−01
rs13065394	G	T	−0.0007	0.0011	5.30E−01	0.0070	0.0012	2.20E−09	0.0002	0.0056	9.74E−01
rs13107325	C	T	−0.0133	0.0018	2.50E−13	−0.0033	0.0020	9.00E−02	−0.0257	0.0096	6.60E−03
rs13255030	A	G	−0.0003	0.0010	8.00E−01	0.0061	0.0011	1.50E−08	−0.0010	0.0052	8.45E−01
rs13269289	G	A	0.0005	0.0010	5.90E−01	−0.0065	0.0011	5.50E−09	−0.0080	0.0055	1.63E−01
rs139911	C	T	−0.0012	0.0010	2.30E−01	0.0088	0.0011	2.20E−16	0.0020	0.0052	7.71E−01
rs1456193	T	C	−0.0032	0.0012	1.20E−02	0.0008	0.0014	5.80E−01	−0.0372	0.0067	2.40E−08
rs1470764	G	A	−0.0001	0.0010	8.80E−01	0.0067	0.0011	5.90E−10	0.0000	0.0052	9.75E−01
rs1494185	G	A	0.0002	0.0011	8.30E−01	0.0067	0.0012	1.10E−08	−0.0020	0.0057	6.80E−01
rs149611468	T	C	−0.0026	0.0045	5.80E−01	0.0281	0.0049	1.30E−08	−0.0218	0.0244	3.84E−01
rs1524472	A	G	0.0002	0.0010	7.90E−01	0.0059	0.0011	2.10E−08	0.0020	0.0051	7.59E−01
rs1607227	G	T	0.0064	0.0011	1.50E−09	−0.0013	0.0012	2.90E−01	0.0119	0.0056	3.46E−02
rs17005118	G	A	−0.0065	0.0011	2.50E−09	0.0014	0.0012	3.10E−01	−0.0266	0.0058	2.85E−06
rs17374439	C	T	−0.0005	0.0012	7.00E−01	−0.0130	0.0013	1.20E−22	0.0024	0.0064	7.03E−01
rs17388803	A	C	−0.0098	0.0016	6.50E−10	0.0024	0.0017	1.80E−01	−0.0178	0.0085	3.76E−02
rs17448682	C	T	0.0028	0.0011	1.40E−02	−0.0071	0.0012	8.30E−09	−0.0040	0.0060	5.31E−01
rs17463545	C	T	−0.0040	0.0018	2.60E−02	−0.0121	0.0019	4.40E−10	0.0002	0.0094	9.82E−01
rs17575798	G	A	0.0007	0.0012	6.00E−01	0.0092	0.0013	7.80E−12	0.0014	0.0064	8.33E−01
rs17682747	G	A	0.0000	0.0011	9.80E−01	−0.0072	0.0013	8.70E−09	0.0181	0.0061	2.85E−03
rs17822102	A	G	0.0003	0.0010	7.90E−01	−0.0070	0.0011	1.30E−10	0.0111	0.0054	3.82E−02
rs1947198	C	T	−0.0014	0.0015	3.60E−01	−0.0093	0.0016	3.20E−09	−0.0020	0.0078	7.56E−01
rs2014830	C	T	0.0058	0.0011	2.70E−08	−0.0019	0.0012	1.10E−01	0.0259	0.0056	3.92E−06
rs202157	C	T	0.0026	0.0011	1.30E−02	0.0093	0.0012	1.30E−15	0.0119	0.0056	2.87E−02
rs205024	C	T	0.0055	0.0010	2.70E−08	−0.0022	0.0011	4.90E−02	0.0118	0.0052	2.43E−02
rs2072727	T	C	−0.0041	0.0010	2.30E−05	0.0065	0.0011	5.60E−10	−0.0112	0.0051	3.02E−02

rs2102506	G	A	−0.0022	0.0010	3.40E−02	0.0069	0.0011	8.30E−10	−0.0102	0.0054	6.15E−02
rs2239626	T	C	0.0010	0.0010	3.10E−01	−0.0079	0.0011	5.00E−12	0.0010	0.0056	8.60E−01
rs231398	G	A	−0.0012	0.0013	3.50E−01	0.0083	0.0014	5.80E−09	0.0004	0.0070	9.53E−01
rs2467109	T	A	0.0019	0.0011	6.20E−02	−0.0065	0.0012	3.90E−08	−0.0040	0.0057	4.67E−01
rs2518022	T	C	−0.0019	0.0017	2.80E−01	0.0147	0.0019	7.60E−15	−0.0083	0.0091	3.60E−01
rs2653343	T	A	0.0048	0.0012	3.70E−05	0.0144	0.0013	5.20E−29	0.0208	0.0062	9.96E−04
rs2820313	A	G	−0.0060	0.0010	2.30E−09	0.0047	0.0011	1.90E−05	−0.0169	0.0054	1.87E−03
rs28380327	A	T	0.0018	0.0010	7.30E−02	0.0068	0.0011	7.80E−10	0.0155	0.0053	3.20E−03
rs2842638	T	G	−0.0026	0.0010	9.00E−03	0.0072	0.0011	1.90E−11	0.0023	0.0053	6.64E−01
rs28634184	C	T	0.0017	0.0011	1.30E−01	0.0070	0.0012	1.30E−08	0.0072	0.0059	2.17E−01
rs2863957	C	A	0.0102	0.0012	2.60E−18	0.0010	0.0013	4.40E−01	0.0182	0.0062	3.22E−03
rs2893787	G	A	−0.0013	0.0011	2.60E−01	0.0068	0.0012	3.50E−08	0.0119	0.0058	4.12E−02
rs2910032	C	T	−0.0031	0.0010	1.40E−03	−0.0079	0.0011	1.10E−13	0.0000	0.0051	9.50E−01
rs2949923	A	G	−0.0006	0.0010	5.50E−01	−0.0062	0.0011	4.20E−09	−0.0109	0.0051	3.69E−02
rs308521	T	C	−0.0015	0.0010	1.30E−01	0.0065	0.0011	1.50E−09	0.0079	0.0052	1.31E−01
rs34581681	G	A	0.0007	0.0013	5.70E−01	0.0081	0.0014	4.20E−08	−0.0040	0.0070	5.28E−01
rs34619169	G	A	0.0005	0.0010	6.40E−01	−0.0064	0.0011	1.50E−08	0.0048	0.0055	3.89E−01
rs34627176	G	A	0.0016	0.0012	1.70E−01	−0.0070	0.0013	3.60E−08	0.0022	0.0063	7.30E−01
rs34875688	T	A	0.0022	0.0011	5.10E−02	0.0075	0.0013	1.10E−09	0.0048	0.0061	4.22E−01
rs35653190	C	T	−0.0008	0.0012	5.10E−01	0.0071	0.0013	2.00E−08	−0.0169	0.0061	6.41E−03
rs370771	G	T	−0.0011	0.0010	2.70E−01	−0.0009	0.0011	4.80E−01	−0.0345	0.0051	1.54E−11
rs3760185	C	T	0.0010	0.0011	3.90E−01	0.0087	0.0012	2.10E−12	−0.0100	0.0060	9.34E−02
rs3767240	T	C	−0.0012	0.0010	2.20E−01	−0.0073	0.0011	7.30E−12	−0.0060	0.0052	2.85E−01
rs3776864	A	C	0.0057	0.0010	1.70E−08	−0.0006	0.0011	5.90E−01	0.0227	0.0054	3.06E−05
rs3850174	T	A	0.0002	0.0011	8.90E−01	0.0075	0.0012	1.40E−09	−0.0020	0.0059	7.10E−01
rs4241964	T	G	−0.0005	0.0010	6.30E−01	−0.0076	0.0011	7.20E−13	−0.0109	0.0051	3.04E−02
rs4321976	T	C	−0.0023	0.0012	4.20E−02	0.0077	0.0013	8.70E−10	−0.0129	0.0061	2.95E−02
rs4339281	A	G	0.0009	0.0014	5.30E−01	0.0092	0.0016	5.30E−09	−0.0149	0.0076	5.45E−02
rs4419127	A	G	−0.0006	0.0010	5.50E−01	0.0080	0.0011	5.70E−13	−0.0010	0.0054	7.93E−01
rs4585442	A	G	−0.0063	0.0010	8.10E−10	0.0023	0.0011	4.80E−02	−0.0257	0.0055	3.09E−06
rs4752593	G	C	0.0009	0.0010	3.90E−01	0.0062	0.0011	9.80E−09	0.0010	0.0053	9.20E−01
rs4822107	G	A	0.0007	0.0010	4.30E−01	−0.0065	0.0011	8.70E−10	0.0040	0.0052	4.55E−01
rs4986172	C	T	−0.0034	0.0010	7.60E−04	−0.0002	0.0011	7.70E−01	−0.0373	0.0054	4.52E−12
rs520954	A	G	0.0004	0.0010	7.60E−01	−0.0099	0.0011	1.60E−18	−0.0040	0.0054	4.27E−01
rs524859	G	A	0.0020	0.0010	5.40E−02	0.0009	0.0011	3.60E−01	0.0308	0.0053	6.83E−09
rs532395	C	T	0.0003	0.0010	7.50E−01	−0.0063	0.0012	2.30E−08	0.0103	0.0056	6.38E−02
rs5757675	G	T	0.0065	0.0011	2.70E−09	−0.0013	0.0012	2.90E−01	0.0100	0.0058	8.92E−02
rs59779556	T	G	0.0055	0.0010	2.00E−08	−0.0026	0.0011	9.60E−03	0.0251	0.0051	9.47E−07
rs60194061	G	A	0.0022	0.0011	4.50E−02	−0.0076	0.0012	8.60E−11	0.0039	0.0058	4.98E−01
rs60616179	A	G	−0.0006	0.0021	8.20E−01	0.0132	0.0023	9.20E−09	0.0062	0.0113	5.84E−01
rs60882754	A	T	0.0113	0.0020	1.80E−08	0.0028	0.0022	2.60E−01	0.0328	0.0107	2.23E−03
rs62553781	C	T	0.0014	0.0026	5.90E−01	0.0194	0.0029	3.00E−11	−0.0060	0.0140	6.61E−01
rs6537834	T	C	−0.0008	0.0010	4.20E−01	−0.0059	0.0011	3.50E−08	−0.0026	0.0052	6.15E−01
rs6561715	T	A	0.0044	0.0010	7.90E−06	−0.0008	0.0011	4.80E−01	0.0373	0.0053	3.67E−12
rs6599694	G	T	−0.0008	0.0010	4.00E−01	0.0062	0.0011	2.10E−08	−0.0070	0.0054	2.14E−01
rs6656331	C	T	−0.0012	0.0010	2.50E−01	0.0064	0.0011	1.50E−09	−0.0032	0.0051	5.35E−01
rs6714679	T	C	0.0030	0.0010	2.70E−03	−0.0017	0.0011	1.40E−01	0.0293	0.0053	3.35E−08
rs6744983	G	T	0.0004	0.0010	6.90E−01	−0.0059	0.0011	4.90E−08	−0.0020	0.0052	7.02E−01
rs6938026	A	G	−0.0043	0.0012	3.20E−04	−0.0026	0.0013	3.60E−02	−0.0373	0.0062	1.70E−09
rs6967481	C	T	0.0009	0.0010	3.40E−01	−0.0070	0.0011	1.90E−11	0.0094	0.0051	6.52E−02
rs7001604	T	C	0.0020	0.0010	3.60E−02	−0.0075	0.0011	7.30E−12	0.0038	0.0053	4.76E−01
rs72632979	A	G	−0.0015	0.0013	2.50E−01	0.0081	0.0014	4.00E−09	0.0015	0.0068	8.31E−01
rs72720396	A	G	0.0043	0.0011	1.60E−04	−0.0101	0.0013	5.30E−16	0.0140	0.0061	2.09E−02
rs72829936	G	A	0.0023	0.0013	7.50E−02	−0.0083	0.0014	5.50E−09	0.0098	0.0069	1.54E−01
rs7302062	T	C	−0.0015	0.0010	1.30E−01	0.0074	0.0011	5.20E−12	0.0015	0.0051	7.71E−01
rs7304278	A	G	−0.0014	0.0011	1.90E−01	−0.0077	0.0012	9.60E−11	−0.0026	0.0057	6.50E−01
rs73606718	G	A	0.0032	0.0015	2.70E−02	0.0094	0.0016	5.70E−09	−0.0070	0.0078	3.93E−01
rs7524118	T	C	−0.0058	0.0011	4.90E−08	−0.0010	0.0012	3.60E−01	−0.0122	0.0056	3.02E−02
rs7547493	A	G	−0.0010	0.0013	4.40E−01	−0.0136	0.0014	2.50E−23	0.0025	0.0067	7.06E−01
rs75650221	C	T	−0.0044	0.0025	7.60E−02	−0.0152	0.0028	3.30E−08	−0.0020	0.0133	8.70E−01
rs769066	T	C	−0.0013	0.0012	2.80E−01	−0.0077	0.0014	1.90E−08	0.0007	0.0066	9.11E−01
rs7691121	C	G	−0.0007	0.0011	5.30E−01	0.0080	0.0012	1.00E−10	−0.0060	0.0060	2.90E−01
rs77008212	A	G	0.0009	0.0017	6.20E−01	0.0193	0.0019	1.10E−24	0.0060	0.0091	5.10E−01
rs7701529	A	T	0.0008	0.0011	4.30E−01	−0.0070	0.0012	1.70E−08	−0.0010	0.0060	8.65E−01
rs77217059	G	A	0.0078	0.0013	6.40E−09	−0.0005	0.0015	7.30E−01	0.0412	0.0072	1.27E−08
rs778147	C	A	0.0001	0.0010	8.70E−01	0.0070	0.0011	4.60E−10	−0.0028	0.0053	5.93E−01

rs7785344	T	C	−0.0024	0.0010	1.30E−02	−0.0059	0.0011	2.60E−08	−0.0134	0.0051	9.43E−03
rs77960	G	A	−0.0031	0.0010	1.90E−03	−0.0062	0.0011	2.30E−08	−0.0334	0.0054	8.82E−10
rs78095690	T	C	0.0008	0.0010	3.40E−01	−0.0065	0.0011	1.10E−09	0.0092	0.0052	7.36E−02
rs786406	A	G	−0.0027	0.0010	8.50E−03	−0.0077	0.0012	1.00E−11	0.0000	0.0056	9.58E−01
rs7959983	T	C	−0.0008	0.0010	4.30E−01	−0.0075	0.0011	4.70E−12	0.0000	0.0052	9.78E−01
rs9321171	C	T	0.0054	0.0010	4.20E−08	0.0005	0.0011	6.10E−01	0.0001	0.0051	9.81E−01
rs9365769	A	G	0.0016	0.0010	1.10E−01	0.0062	0.0011	7.30E−09	0.0129	0.0052	1.46E−02
rs9369915	G	A	0.0014	0.0010	2.00E−01	−0.0087	0.0012	3.00E−14	0.0072	0.0056	1.93E−01
rs9573971	A	G	0.0002	0.0026	9.50E−01	0.0254	0.0029	4.90E−18	0.0054	0.0141	7.02E−01
rs957501	T	A	−0.0007	0.0010	4.60E−01	0.0062	0.0011	3.00E−08	0.0030	0.0054	5.63E−01
rs9576155	G	A	−0.0025	0.0010	1.40E−02	−0.0022	0.0011	4.50E−02	−0.0315	0.0054	8.00E−09
rs9597241	A	C	0.0013	0.0012	3.10E−01	0.0076	0.0014	2.70E−08	−0.0139	0.0065	2.73E−02

Table S4. Genetical instrumental variables used in the mediation MR analysis

NO	SNP	Effect Allele	Other Allele	Exposure					Outcome				
				Beta	SE	P	Exposure	Sample Size	Beta	SE	P	Outcome	Sample Size
1	rs11763750	G	A	0.0072	0.0012	5.10E−09	Duration Short	411934	0.0207	0.0091	0.0229	Atrial fibrillation	588190
2	rs1229762	C	T	−0.0072	0.0010	1.00E−12	Duration Short	411934	−0.0060	0.0079	0.4494	Atrial fibrillation	588190
3	rs12518468	T	C	−0.0059	0.0010	8.50E−09	Duration Short	411934	0.0035	0.0076	0.6475	Atrial fibrillation	588190
4	rs12567114	G	A	0.0063	0.0011	4.10E−09	Duration Short	411934	−0.0005	0.0080	0.9478	Atrial fibrillation	588190
5	rs12661667	C	T	−0.0060	0.0011	2.80E−08	Duration Short	411934	−0.0021	0.0082	0.7937	Atrial fibrillation	588190
6	rs12963463	C	T	0.0071	0.0011	1.90E−11	Duration Short	411934	0.0066	0.0078	0.3935	Atrial fibrillation	588190
7	rs13107325	C	T	−0.0133	0.0018	2.50E−13	Duration Short	411934	−0.0003	0.0149	0.9856	Atrial fibrillation	588190
8	rs1380703	A	G	−0.0068	0.0010	1.60E−11	Duration Short	411934	−0.0019	0.0077	0.8024	Atrial fibrillation	588190
9	rs142180737	T	C	−0.0309	0.0053	4.40E−09	Duration Short	411934	−0.0162	0.0580	0.7794	Atrial fibrillation	588190
10	rs1607227	G	T	0.0064	0.0011	1.50E−09	Duration Short	411934	0.0094	0.0079	0.2367	Atrial fibrillation	588190
11	rs17005118	G	A	−0.0065	0.0011	2.50E−09	Duration Short	411934	−0.0154	0.0080	0.0564	Atrial fibrillation	588190
12	rs17388803	A	C	−0.0098	0.0016	6.50E−10	Duration Short	411934	0.0050	0.0122	0.6801	Atrial fibrillation	588190
13	rs2014830	C	T	0.0058	0.0011	2.70E−08	Duration Short	411934	0.0120	0.0078	0.1235	Atrial fibrillation	588190
14	rs205024	C	T	0.0055	0.0010	2.70E−08	Duration Short	411934	0.0009	0.0073	0.9057	Atrial fibrillation	588190
15	rs2820313	A	G	−0.0060	0.0010	2.30E−09	Duration Short	411934	−0.0157	0.0077	0.0404	Atrial fibrillation	588190
16	rs2863957	C	A	0.0102	0.0012	2.60E−18	Duration Short	411934	−0.0022	0.0087	0.7963	Atrial fibrillation	588190
17	rs3776864	A	C	0.0057	0.0010	1.70E−08	Duration Short	411934	−0.0132	0.0077	0.0879	Atrial fibrillation	588190
18	rs4585442	A	G	−0.0063	0.0010	8.10E−10	Duration Short	411934	0.0113	0.0077	0.1427	Atrial fibrillation	588190
19	rs5757675	G	T	0.0065	0.0011	2.70E−09	Duration Short	411934	0.0201	0.0083	0.0155	Atrial fibrillation	588190
20	rs59779556	T	G	0.0055	0.0010	2.00E−08	Duration Short	411934	−0.0044	0.0071	0.5389	Atrial fibrillation	588190
21	rs60882754	A	T	0.0113	0.0020	1.80E−08	Duration Short	411934	0.0132	0.0160	0.4088	Atrial fibrillation	588190
22	rs7524118	T	C	−0.0058	0.0011	4.90E−08	Duration Short	411934	0.0064	0.0078	0.4157	Atrial fibrillation	588190
23	rs7939345	T	G	0.0065	0.0012	4.00E−08	Duration Short	411934	−0.0062	0.0091	0.4932	Atrial fibrillation	588190
24	rs9321171	C	T	0.0054	0.0010	4.20E−08	Duration Short	411934	0.0029	0.0073	0.6918	Atrial fibrillation	588190
1	rs1229762	C	T	−0.0072	0.0010	1.00E−12	Duration Short	411934	−0.0038	0.0018	0.0390	BMI	692163
2	rs12661667	C	T	−0.0060	0.0011	2.80E−08	Duration Short	411934	−0.0001	0.0019	0.9700	BMI	692334
3	rs17005118	G	A	−0.0065	0.0011	2.50E−09	Duration Short	411934	−0.0009	0.0018	0.6100	BMI	793499
4	rs17285320	G	T	0.0110	0.0020	5.30E−08	Duration Short	411934	−0.0044	0.0037	0.2300	BMI	691604
5	rs17388803	A	C	−0.0098	0.0016	6.50E−10	Duration Short	411934	0.0008	0.0029	0.7800	BMI	668775
6	rs1823125	A	G	0.0100	0.0012	9.40E−18	Duration Short	411934	0.0042	0.0021	0.0430	BMI	692112
7	rs205024	C	T	0.0055	0.0010	2.70E−08	Duration Short	411934	0.0020	0.0018	0.2700	BMI	691846
8	rs7524118	T	C	−0.0058	0.0011	4.90E−08	Duration Short	411934	0.0004	0.0019	0.8500	BMI	691736

9	rs9929277	A	G	0.0052	0.0010	1.00E−07	Duration Short	411934	0.0046	0.0017	0.0072	BMI	692473
1	rs11763750	G	A	0.0072	0.0012	5.10E−09	Duration Short	411934	0.0277	0.0070	0.0001	CAD	547261
2	rs1229762	C	T	−0.0072	0.0010	1.00E−12	Duration Short	411934	−0.0239	0.0062	0.0001	CAD	547261
3	rs12518468	T	C	−0.0059	0.0010	8.50E−09	Duration Short	411934	0.0020	0.0061	0.7393	CAD	547261
4	rs12567114	G	A	0.0063	0.0011	4.10E−09	Duration Short	411934	−0.0008	0.0064	0.8938	CAD	547261
5	rs12661667	C	T	−0.0060	0.0011	2.80E−08	Duration Short	411934	0.0061	0.0065	0.3462	CAD	547261
6	rs12963463	C	T	0.0071	0.0011	1.90E−11	Duration Short	411934	0.0026	0.0062	0.6764	CAD	547261
7	rs13107325	C	T	−0.0133	0.0018	2.50E−13	Duration Short	411934	0.0003	0.0112	0.9759	CAD	547261
8	rs1380703	A	G	−0.0068	0.0010	1.60E−11	Duration Short	411934	0.0005	0.0060	0.9378	CAD	547261
9	rs1607227	G	T	0.0064	0.0011	1.50E−09	Duration Short	411934	0.0041	0.0062	0.5055	CAD	547261
10	rs17005118	G	A	−0.0065	0.0011	2.50E−09	Duration Short	411934	−0.0176	0.0057	0.0021	CAD	547261
11	rs17388803	A	C	−0.0098	0.0016	6.50E−10	Duration Short	411934	0.0043	0.0093	0.6479	CAD	547261
12	rs2014830	C	T	0.0058	0.0011	2.70E−08	Duration Short	411934	0.0116	0.0056	0.0398	CAD	547261
13	rs205024	C	T	0.0055	0.0010	2.70E−08	Duration Short	411934	0.0005	0.0058	0.9312	CAD	547261
14	rs2863957	C	A	0.0102	0.0012	2.60E−18	Duration Short	411934	0.0020	0.0068	0.7685	CAD	547261
15	rs3776864	A	C	0.0057	0.0010	1.70E−08	Duration Short	411934	0.0076	0.0062	0.2244	CAD	547261
16	rs4585442	A	G	−0.0063	0.0010	8.10E−10	Duration Short	411934	−0.0053	0.0061	0.3814	CAD	547261
17	rs5757675	G	T	0.0065	0.0011	2.70E−09	Duration Short	411934	0.0132	0.0065	0.0432	CAD	547261
18	rs59779556	T	G	0.0055	0.0010	2.00E−08	Duration Short	411934	0.0043	0.0056	0.4424	CAD	547261
19	rs60882754	A	T	0.0113	0.0020	1.80E−08	Duration Short	411934	0.0284	0.0120	0.0183	CAD	547261
20	rs7524118	T	C	−0.0058	0.0011	4.90E−08	Duration Short	411934	−0.0006	0.0061	0.9255	CAD	547261
21	rs7939345	T	G	0.0065	0.0012	4.00E−08	Duration Short	411934	−0.0007	0.0069	0.9226	CAD	547261
22	rs9321171	C	T	0.0054	0.0010	4.20E−08	Duration Short	411934	−0.0014	0.0057	0.8101	CAD	547261
1	rs11763750	G	A	0.0072	0.0012	5.10E−09	Duration Short	411934	0.0226	0.0100	0.0232	Heart failure	958797
2	rs1229762	C	T	−0.0072	0.0010	1.00E−12	Duration Short	411934	−0.0047	0.0083	0.5740	Heart failure	963989
3	rs12518468	T	C	−0.0059	0.0010	8.50E−09	Duration Short	411934	−0.0006	0.0084	0.9447	Heart failure	962013
4	rs12567114	G	A	0.0063	0.0011	4.10E−09	Duration Short	411934	−0.0069	0.0088	0.4362	Heart failure	951827
5	rs12661667	C	T	−0.0060	0.0011	2.80E−08	Duration Short	411934	0.0126	0.0090	0.1615	Heart failure	952643
6	rs12963463	C	T	0.0071	0.0011	1.90E−11	Duration Short	411934	0.0113	0.0087	0.1935	Heart failure	963756
7	rs13107325	C	T	−0.0133	0.0018	2.50E−13	Duration Short	411934	−0.0701	0.0170	0.0000	Heart failure	952649
8	rs1380703	A	G	−0.0068	0.0010	1.60E−11	Duration Short	411934	0.0016	0.0083	0.8463	Heart failure	958071
9	rs1607227	G	T	0.0064	0.0011	1.50E−09	Duration Short	411934	0.0037	0.0086	0.6712	Heart failure	956725
10	rs17005118	G	A	−0.0065	0.0011	2.50E−09	Duration Short	411934	−0.0174	0.0088	0.0478	Heart failure	960987
11	rs17388803	A	C	−0.0098	0.0016	6.50E−10	Duration Short	411934	0.0071	0.0125	0.5706	Heart failure	961954
12	rs2014830	C	T	0.0058	0.0011	2.70E−08	Duration Short	411934	0.0087	0.0085	0.3055	Heart failure	960774
13	rs205024	C	T	0.0055	0.0010	2.70E−08	Duration Short	411934	0.0113	0.0080	0.1587	Heart failure	964027

14	rs2820313	A	G	−0.0060	0.0010	2.30E−09	Duration Short	411934	−0.0122	0.0083	0.1420	Heart failure	955739
15	rs2863957	C	A	0.0102	0.0012	2.60E−18	Duration Short	411934	0.0112	0.0095	0.2409	Heart failure	948821
16	rs3776864	A	C	0.0057	0.0010	1.70E−08	Duration Short	411934	0.0021	0.0085	0.8018	Heart failure	964010
17	rs4585442	A	G	−0.0063	0.0010	8.10E−10	Duration Short	411934	−0.0019	0.0085	0.8280	Heart failure	961999
18	rs5757675	G	T	0.0065	0.0011	2.70E−09	Duration Short	411934	0.0013	0.0089	0.8826	Heart failure	958742
19	rs59779556	T	G	0.0055	0.0010	2.00E−08	Duration Short	411934	−0.0015	0.0079	0.8481	Heart failure	958780
20	rs60882754	A	T	0.0113	0.0020	1.80E−08	Duration Short	411934	−0.0076	0.0178	0.6678	Heart failure	958804
21	rs7524118	T	C	−0.0058	0.0011	4.90E−08	Duration Short	411934	0.0014	0.0085	0.8658	Heart failure	959013
22	rs7939345	T	G	0.0065	0.0012	4.00E−08	Duration Short	411934	−0.0003	0.0100	0.9797	Heart failure	953342
23	rs9321171	C	T	0.0054	0.0010	4.20E−08	Duration Short	411934	−0.0058	0.0079	0.4611	Heart failure	955708
1	rs11763750	G	A	0.0072	0.0012	5.10E−09	Duration Short	411934	0.0116	0.0127	0.3598	AIS	440328
2	rs1229762	C	T	−0.0072	0.0010	1.00E−12	Duration Short	411934	0.0005	0.0105	0.9608	AIS	440328
3	rs12518468	T	C	−0.0059	0.0010	8.50E−09	Duration Short	411934	−0.0007	0.0109	0.9525	AIS	440328
4	rs12567114	G	A	0.0063	0.0011	4.10E−09	Duration Short	411934	−0.0061	0.0113	0.5881	AIS	440328
5	rs12661667	C	T	−0.0060	0.0011	2.80E−08	Duration Short	411934	0.0145	0.0113	0.2000	AIS	440328
6	rs12963463	C	T	0.0071	0.0011	1.90E−11	Duration Short	411934	−0.0086	0.0111	0.4357	AIS	440328
7	rs13107325	C	T	−0.0133	0.0018	2.50E−13	Duration Short	411934	0.0065	0.0215	0.7611	AIS	440328
8	rs1380703	A	G	−0.0068	0.0010	1.60E−11	Duration Short	411934	−0.0040	0.0106	0.7088	AIS	440328
9	rs1607227	G	T	0.0064	0.0011	1.50E−09	Duration Short	411934	−0.0050	0.0111	0.6541	AIS	440328
10	rs17005118	G	A	−0.0065	0.0011	2.50E−09	Duration Short	411934	−0.0120	0.0112	0.2863	AIS	440328
11	rs17388803	A	C	−0.0098	0.0016	6.50E−10	Duration Short	411934	−0.0317	0.0157	0.0438	AIS	440328
12	rs2014830	C	T	0.0058	0.0011	2.70E−08	Duration Short	411934	0.0119	0.0109	0.2742	AIS	440328
13	rs205024	C	T	0.0055	0.0010	2.70E−08	Duration Short	411934	0.0104	0.0104	0.3163	AIS	440328
14	rs2820313	A	G	−0.0060	0.0010	2.30E−09	Duration Short	411934	−0.0171	0.0105	0.1039	AIS	440328
15	rs2863957	C	A	0.0102	0.0012	2.60E−18	Duration Short	411934	0.0027	0.0120	0.8223	AIS	440328
16	rs3776864	A	C	0.0057	0.0010	1.70E−08	Duration Short	411934	−0.0026	0.0110	0.8138	AIS	440328
17	rs4585442	A	G	−0.0063	0.0010	8.10E−10	Duration Short	411934	−0.0034	0.0107	0.7466	AIS	440328
18	rs5757675	G	T	0.0065	0.0011	2.70E−09	Duration Short	411934	0.0024	0.0114	0.8359	AIS	440328
19	rs59779556	T	G	0.0055	0.0010	2.00E−08	Duration Short	411934	−0.0014	0.0101	0.8901	AIS	440328
20	rs60882754	A	T	0.0113	0.0020	1.80E−08	Duration Short	411934	0.0092	0.0233	0.6928	AIS	440328
21	rs7524118	T	C	−0.0058	0.0011	4.90E−08	Duration Short	411934	0.0151	0.0111	0.1734	AIS	440328
22	rs7939345	T	G	0.0065	0.0012	4.00E−08	Duration Short	411934	0.0020	0.0127	0.8775	AIS	440328
23	rs9321171	C	T	0.0054	0.0010	4.20E−08	Duration Short	411934	0.0013	0.0098	0.8906	AIS	440328
1	rs11763750	G	A	0.0072	0.0012	5.10E−09	Duration Short	411934	0.0250	0.0093	0.0063	T2D	157384
2	rs1229762	C	T	−0.0072	0.0010	1.00E−12	Duration Short	411934	−0.0150	0.0079	0.0650	T2D	157384
3	rs12518468	T	C	−0.0059	0.0010	8.50E−09	Duration Short	411934	0.0071	0.0079	0.3700	T2D	157384

4	rs12567114	G	A	0.0063	0.0011	4.10E−09	Duration Short	411934	0.0049	0.0084	0.5600	T2D	157384
5	rs12661667	C	T	−0.0060	0.0011	2.80E−08	Duration Short	411934	0.0019	0.0085	0.8200	T2D	157384
6	rs12963463	C	T	0.0071	0.0011	1.90E−11	Duration Short	411934	−0.0087	0.0083	0.2900	T2D	157384
7	rs13107325	C	T	−0.0133	0.0018	2.50E−13	Duration Short	411934	−0.0018	0.0150	0.9000	T2D	157384
8	rs1380703	A	G	−0.0068	0.0010	1.60E−11	Duration Short	411934	0.0098	0.0078	0.2100	T2D	157384
9	rs142180737	T	C	−0.0309	0.0053	4.40E−09	Duration Short	411934	−0.1000	0.0470	0.0280	T2D	156272
10	rs1607227	G	T	0.0064	0.0011	1.50E−09	Duration Short	411934	−0.0008	0.0082	0.9200	T2D	157384
11	rs17005118	G	A	−0.0065	0.0011	2.50E−09	Duration Short	411934	−0.0084	0.0085	0.3200	T2D	157384
12	rs17388803	A	C	−0.0098	0.0016	6.50E−10	Duration Short	411934	−0.0037	0.0120	0.7600	T2D	157384
13	rs2014830	C	T	0.0058	0.0011	2.70E−08	Duration Short	411934	0.0240	0.0081	0.0027	T2D	157384
14	rs205024	C	T	0.0055	0.0010	2.70E−08	Duration Short	411934	0.0095	0.0076	0.2100	T2D	157384
15	rs2820313	A	G	−0.0060	0.0010	2.30E−09	Duration Short	411934	−0.0110	0.0079	0.1500	T2D	157384
16	rs2863957	C	A	0.0102	0.0012	2.60E−18	Duration Short	411934	−0.0085	0.0090	0.3400	T2D	157384
17	rs4585442	A	G	−0.0063	0.0010	8.10E−10	Duration Short	411934	−0.0095	0.0081	0.2400	T2D	157384
18	rs5757675	G	T	0.0065	0.0011	2.70E−09	Duration Short	411934	0.0220	0.0084	0.0088	T2D	157384
19	rs59779556	T	G	0.0055	0.0010	2.00E−08	Duration Short	411934	0.0079	0.0075	0.2900	T2D	157384
20	rs60882754	A	T	0.0113	0.0020	1.80E−08	Duration Short	411934	−0.0092	0.0160	0.5700	T2D	157384
21	rs7524118	T	C	−0.0058	0.0011	4.90E−08	Duration Short	411934	−0.0069	0.0081	0.3900	T2D	157384
22	rs7939345	T	G	0.0065	0.0012	4.00E−08	Duration Short	411934	0.0190	0.0092	0.0420	T2D	157073
23	rs9321171	C	T	0.0054	0.0010	4.20E−08	Duration Short	411934	−0.0036	0.0075	0.6300	T2D	157384
1	rs1229762	C	T	−0.0072	0.0010	1.00E−12	Duration Short	411934	−0.0116	0.0046	0.0111	Depression	500199
2	rs12518468	T	C	−0.0059	0.0010	8.50E−09	Duration Short	411934	−0.0097	0.0046	0.0349	Depression	500199
3	rs12567114	G	A	0.0063	0.0011	4.10E−09	Duration Short	411934	0.0106	0.0050	0.0331	Depression	500199
4	rs12661667	C	T	−0.0060	0.0011	2.80E−08	Duration Short	411934	−0.0070	0.0049	0.1539	Depression	500199
5	rs13107325	C	T	−0.0133	0.0018	2.50E−13	Duration Short	411934	−0.0242	0.0084	0.0039	Depression	500199
6	rs142180737	T	C	−0.0309	0.0053	4.40E−09	Duration Short	411934	−0.0635	0.0272	0.0195	Depression	500199
7	rs1607227	G	T	0.0064	0.0011	1.50E−09	Duration Short	411934	0.0145	0.0047	0.0021	Depression	500199
8	rs17005118	G	A	−0.0065	0.0011	2.50E−09	Duration Short	411934	0.0068	0.0049	0.1648	Depression	500199
9	rs17388803	A	C	−0.0098	0.0016	6.50E−10	Duration Short	411934	−0.0024	0.0070	0.7348	Depression	500199
10	rs2014830	C	T	0.0058	0.0011	2.70E−08	Duration Short	411934	0.0123	0.0047	0.0092	Depression	500199
11	rs205024	C	T	0.0055	0.0010	2.70E−08	Duration Short	411934	0.0135	0.0044	0.0023	Depression	500199
12	rs2820313	A	G	−0.0060	0.0010	2.30E−09	Duration Short	411934	−0.0035	0.0045	0.4375	Depression	500199
13	rs2863957	C	A	0.0102	0.0012	2.60E−18	Duration Short	411934	0.0020	0.0052	0.6976	Depression	500199
14	rs3776864	A	C	0.0057	0.0010	1.70E−08	Duration Short	411934	0.0035	0.0046	0.4424	Depression	500199
15	rs4585442	A	G	−0.0063	0.0010	8.10E−10	Duration Short	411934	−0.0069	0.0047	0.1374	Depression	500199
16	rs5757675	G	T	0.0065	0.0011	2.70E−09	Duration Short	411934	−0.0056	0.0049	0.2507	Depression	500199

17	rs59779556	T	G	0.0055	0.0010	2.00E−08	Duration Short	411934	0.0011	0.0043	0.8061	Depression	500199
18	rs60882754	A	T	0.0113	0.0020	1.80E−08	Duration Short	411934	0.0049	0.0091	0.5910	Depression	500199
19	rs7939345	T	G	0.0065	0.0012	4.00E−08	Duration Short	411934	0.0200	0.0053	0.0002	Depression	500199
20	rs9321171	C	T	0.0054	0.0010	4.20E−08	Duration Short	411934	−0.0029	0.0043	0.5054	Depression	500199
1	rs10093110	A	G	−0.0319	0.0057	1.88E−08	CAD	547261	0.0070	0.0039	0.0749	Lifespan	640154
2	rs10237377	T	G	−0.0338	0.0058	6.53E−09	CAD	547261	0.0050	0.0041	0.2194	Lifespan	640187
3	rs10267593	A	G	−0.0360	0.0064	1.88E−08	CAD	547261	0.0121	0.0051	0.0179	Lifespan	640185
4	rs10456100	T	C	0.0396	0.0063	3.61E−10	CAD	547261	−0.0116	0.0043	0.0067	Lifespan	638067
5	rs10512861	T	G	−0.0431	0.0073	3.00E−09	CAD	547261	0.0160	0.0057	0.0048	Lifespan	640187
6	rs10841443	C	G	−0.0460	0.0061	2.86E−14	CAD	547261	0.0119	0.0042	0.0044	Lifespan	635243
7	rs10857147	A	T	−0.0491	0.0064	1.37E−14	CAD	547261	0.0131	0.0043	0.0026	Lifespan	637314
8	rs10858079	A	G	0.0365	0.0065	2.02E−08	CAD	547261	0.0083	0.0050	0.0952	Lifespan	640165
9	rs11057830	A	G	0.0655	0.0075	1.91E−18	CAD	547261	−0.0213	0.0056	0.0002	Lifespan	640187
10	rs11080107	T	C	−0.0358	0.0057	2.90E−10	CAD	547261	0.0042	0.0039	0.2847	Lifespan	637314
11	rs11099493	A	G	0.0390	0.0062	2.49E−10	CAD	547261	−0.0076	0.0042	0.0683	Lifespan	640185
12	rs11107903	A	G	−0.0747	0.0105	1.06E−12	CAD	547261	0.0289	0.0075	0.0001	Lifespan	638110
13	rs11170820	C	G	−0.0832	0.0117	9.28E−13	CAD	547261	0.0242	0.0086	0.0049	Lifespan	637314
14	rs112941079	A	G	0.0615	0.0085	3.75E−13	CAD	547261	−0.0171	0.0056	0.0021	Lifespan	628619
15	rs11556924	T	C	−0.0548	0.0055	1.37E−23	CAD	547261	0.0202	0.0040	0.0000	Lifespan	637307
16	rs11591147	T	G	−0.2406	0.0247	1.86E−22	CAD	547261	0.0477	0.0153	0.0019	Lifespan	623878
17	rs11601507	A	C	0.0782	0.0108	5.61E−13	CAD	547261	−0.0208	0.0077	0.0067	Lifespan	637314
18	rs11652858	T	C	−0.0352	0.0060	4.03E−09	CAD	547261	0.0090	0.0041	0.0289	Lifespan	640165
19	rs11673093	A	G	0.0429	0.0065	5.22E−11	CAD	547261	−0.0187	0.0045	0.0000	Lifespan	628619
20	rs11677932	A	G	−0.0339	0.0061	2.64E−08	CAD	547261	−0.0014	0.0042	0.7392	Lifespan	640187
21	rs1169288	A	C	−0.0490	0.0056	1.26E−18	CAD	547261	0.0157	0.0042	0.0002	Lifespan	626548
22	rs12143614	A	T	0.0570	0.0094	1.29E−09	CAD	547261	−0.0021	0.0062	0.7366	Lifespan	637315
23	rs12149545	A	G	−0.0374	0.0062	1.19E−09	CAD	547261	0.0121	0.0042	0.0037	Lifespan	640176
24	rs12212146	T	C	0.0915	0.0124	1.71E−13	CAD	547261	−0.0089	0.0078	0.2525	Lifespan	637314
25	rs12500824	A	G	0.0336	0.0053	1.64E−10	CAD	547261	−0.0133	0.0040	0.0010	Lifespan	640153
26	rs1250229	T	C	0.0435	0.0059	1.58E−13	CAD	547261	−0.0113	0.0044	0.0109	Lifespan	635243
27	rs12801636	A	G	−0.0403	0.0060	2.29E−11	CAD	547261	0.0121	0.0046	0.0091	Lifespan	640186
28	rs12897	A	G	−0.0358	0.0059	1.21E−09	CAD	547261	0.0014	0.0040	0.7358	Lifespan	640185
29	rs12936587	A	G	−0.0320	0.0052	9.51E−10	CAD	547261	0.0045	0.0039	0.2493	Lifespan	635243
30	rs12999907	A	G	0.0482	0.0074	6.31E−11	CAD	547261	0.0039	0.0051	0.4462	Lifespan	638091
31	rs1317507	A	C	0.0398	0.0058	8.21E−12	CAD	547261	−0.0190	0.0044	0.0000	Lifespan	636850
32	rs140570886	T	C	−0.4778	0.0236	2.06E−91	CAD	547261	0.1274	0.0163	0.0000	Lifespan	626563

33	rs1412444	T	C	0.0559	0.0059	2.43E−21	CAD	547261	−0.0065	0.0041	0.1090	Lifespan	640187
34	rs1495746	T	C	−0.0352	0.0063	2.47E−08	CAD	547261	0.0093	0.0043	0.0308	Lifespan	640187
35	rs1591805	A	G	0.0370	0.0058	1.17E−10	CAD	547261	0.0029	0.0039	0.4560	Lifespan	635245
36	rs16823332	T	C	−0.0496	0.0090	3.65E−08	CAD	547261	0.0004	0.0061	0.9446	Lifespan	640153
37	rs16986953	A	G	0.0812	0.0098	1.06E−16	CAD	547261	−0.0170	0.0076	0.0254	Lifespan	640187
38	rs170041	T	C	−0.0469	0.0056	4.11E−17	CAD	547261	0.0085	0.0043	0.0468	Lifespan	640185
39	rs17080091	T	C	−0.0537	0.0092	6.09E−09	CAD	547261	0.0293	0.0076	0.0001	Lifespan	638117
40	rs17081933	A	T	0.0399	0.0070	1.17E−08	CAD	547261	−0.0110	0.0049	0.0263	Lifespan	640187
41	rs17091891	T	C	0.0585	0.0078	5.15E−14	CAD	547261	−0.0206	0.0060	0.0006	Lifespan	625081
42	rs17114046	A	G	0.0960	0.0088	8.04E−28	CAD	547261	−0.0250	0.0067	0.0002	Lifespan	633089
43	rs17478367	C	G	0.0458	0.0081	1.94E−08	CAD	547261	−0.0101	0.0058	0.0801	Lifespan	635243
44	rs17514846	A	C	0.0559	0.0052	9.86E−27	CAD	547261	−0.0247	0.0039	0.0000	Lifespan	640187
45	rs17616620	A	G	0.0568	0.0098	6.01E−09	CAD	547261	−0.0269	0.0071	0.0002	Lifespan	637314
46	rs17680741	T	C	0.0420	0.0062	1.72E−11	CAD	547261	−0.0004	0.0043	0.9320	Lifespan	638108
47	rs1807214	A	C	0.0639	0.0097	5.21E−11	CAD	547261	−0.0043	0.0074	0.5638	Lifespan	636177
48	rs184278183	T	C	0.3370	0.0323	1.92E−25	CAD	547261	−0.1271	0.0229	0.0000	Lifespan	389513
49	rs185244	T	C	0.0644	0.0076	2.40E−17	CAD	547261	−0.0118	0.0053	0.0244	Lifespan	631477
50	rs1870634	T	G	−0.0600	0.0059	3.50E−24	CAD	547261	0.0079	0.0041	0.0525	Lifespan	640181
51	rs1892094	T	C	−0.0362	0.0052	3.14E−12	CAD	547261	0.0098	0.0039	0.0109	Lifespan	640187
52	rs2107595	A	G	0.0752	0.0073	1.25E−24	CAD	547261	−0.0264	0.0053	0.0000	Lifespan	638100
53	rs2107732	A	G	−0.0567	0.0103	3.64E−08	CAD	547261	0.0103	0.0067	0.1278	Lifespan	637314
54	rs2145598	A	G	−0.0283	0.0052	4.26E−08	CAD	547261	0.0034	0.0039	0.3837	Lifespan	640185
55	rs2189839	A	G	0.0345	0.0063	4.53E−08	CAD	547261	−0.0144	0.0042	0.0007	Lifespan	638116
56	rs2252641	T	C	−0.0368	0.0051	5.41E−13	CAD	547261	0.0007	0.0039	0.8654	Lifespan	640189
57	rs2327429	T	C	0.0655	0.0056	3.56E−31	CAD	547261	−0.0090	0.0043	0.0366	Lifespan	637314
58	rs246600	T	C	0.0430	0.0051	6.53E−17	CAD	547261	−0.0057	0.0039	0.1428	Lifespan	640153
59	rs2493298	A	C	0.0514	0.0084	9.97E−10	CAD	547261	−0.0134	0.0056	0.0170	Lifespan	637314
60	rs2519093	T	C	0.0554	0.0072	2.03E−14	CAD	547261	−0.0224	0.0050	0.0000	Lifespan	635499
61	rs2571445	A	G	0.0370	0.0052	1.56E−12	CAD	547261	−0.0042	0.0039	0.2844	Lifespan	640179
62	rs260020	T	C	0.0518	0.0084	7.96E−10	CAD	547261	−0.0159	0.0059	0.0070	Lifespan	628282
63	rs2681492	T	C	−0.0558	0.0072	8.64E−15	CAD	547261	−0.0026	0.0052	0.6136	Lifespan	624084
64	rs273909	A	G	−0.0488	0.0078	4.74E−10	CAD	547261	0.0116	0.0060	0.0542	Lifespan	640187
65	rs2814944	A	G	0.0479	0.0072	2.15E−11	CAD	547261	−0.0213	0.0054	0.0001	Lifespan	639160
66	rs2820315	T	C	0.0359	0.0056	1.10E−10	CAD	547261	−0.0041	0.0042	0.3193	Lifespan	640169
67	rs28451064	A	G	0.1083	0.0090	2.58E−33	CAD	547261	−0.0094	0.0059	0.1118	Lifespan	637314
68	rs34991912	T	C	0.0379	0.0057	3.21E−11	CAD	547261	−0.0087	0.0039	0.0270	Lifespan	638072

69	rs35158675	A	G	0.0699	0.0064	5.91E−28	CAD	547261	−0.0028	0.0045	0.5285	Lifespan	579394
70	rs36096196	T	C	0.0469	0.0082	1.33E−08	CAD	547261	−0.0087	0.0056	0.1178	Lifespan	635243
71	rs3740390	T	C	−0.0662	0.0084	4.67E−15	CAD	547261	0.0055	0.0070	0.4363	Lifespan	640181
72	rs3775058	A	T	0.0386	0.0067	7.63E−09	CAD	547261	−0.0130	0.0047	0.0059	Lifespan	640171
73	rs3783324	T	C	−0.0408	0.0071	7.85E−09	CAD	547261	0.0160	0.0050	0.0013	Lifespan	640183
74	rs3809346	A	G	0.0440	0.0053	8.51E−17	CAD	547261	−0.0064	0.0039	0.1032	Lifespan	628953
75	rs3827066	T	C	0.0424	0.0072	4.40E−09	CAD	547261	−0.0161	0.0054	0.0031	Lifespan	640183
76	rs3918226	T	C	0.1071	0.0115	1.35E−20	CAD	547261	−0.0330	0.0073	0.0000	Lifespan	637314
77	rs3936511	A	G	−0.0366	0.0067	3.74E−08	CAD	547261	0.0090	0.0049	0.0668	Lifespan	640143
78	rs4072980	A	G	−0.0326	0.0052	4.13E−10	CAD	547261	0.0002	0.0040	0.9629	Lifespan	637314
79	rs4368453	T	C	0.0342	0.0061	1.81E−08	CAD	547261	−0.0155	0.0042	0.0002	Lifespan	640185
80	rs4613862	A	C	0.0319	0.0052	6.58E−10	CAD	547261	−0.0015	0.0039	0.7014	Lifespan	640179
81	rs4678145	C	G	0.0639	0.0083	1.45E−14	CAD	547261	−0.0077	0.0057	0.1776	Lifespan	624084
82	rs4724806	C	G	0.0386	0.0069	2.26E−08	CAD	547261	−0.0141	0.0046	0.0022	Lifespan	635243
83	rs4752700	A	G	−0.0332	0.0051	8.02E−11	CAD	547261	−0.0013	0.0039	0.7308	Lifespan	640181
84	rs4803455	A	C	−0.0484	0.0057	2.36E−17	CAD	547261	0.0056	0.0039	0.1498	Lifespan	640050
85	rs4845625	T	C	0.0395	0.0051	6.69E−15	CAD	547261	−0.0180	0.0039	0.0000	Lifespan	640162
86	rs4918065	T	C	−0.0372	0.0065	1.22E−08	CAD	547261	0.0120	0.0045	0.0077	Lifespan	624038
87	rs4977574	A	G	−0.1788	0.0056	1.00E−200	CAD	547261	0.0249	0.0039	0.0000	Lifespan	640189
88	rs515135	T	C	−0.0555	0.0066	5.74E−17	CAD	547261	0.0123	0.0050	0.0142	Lifespan	640177
89	rs55730499	T	C	0.3122	0.0118	9.78E−154	CAD	547261	−0.0760	0.0074	0.0000	Lifespan	635243
90	rs55791371	A	C	0.1157	0.0092	1.93E−36	CAD	547261	−0.0339	0.0061	0.0000	Lifespan	635243
91	rs6001960	A	G	0.0351	0.0064	4.40E−08	CAD	547261	−0.0049	0.0045	0.2700	Lifespan	631490
92	rs602633	T	G	−0.0999	0.0062	3.63E−58	CAD	547261	0.0218	0.0047	0.0000	Lifespan	637314
93	rs606452	A	C	−0.0466	0.0071	4.76E−11	CAD	547261	0.0084	0.0056	0.1329	Lifespan	626155
94	rs607562	T	G	−0.0377	0.0058	1.08E−10	CAD	547261	0.0155	0.0044	0.0004	Lifespan	638107
95	rs6102343	A	G	0.0372	0.0065	1.12E−08	CAD	547261	−0.0071	0.0045	0.1178	Lifespan	640179
96	rs61848342	T	C	−0.0363	0.0059	6.38E−10	CAD	547261	0.0021	0.0040	0.6087	Lifespan	633233
97	rs62076439	T	G	0.0480	0.0060	1.63E−15	CAD	547261	−0.0075	0.0040	0.0606	Lifespan	638102
98	rs62233066	A	G	0.1063	0.0180	3.73E−09	CAD	547261	0.0150	0.0190	0.4300	Lifespan	569081
99	rs644045	A	G	−0.0413	0.0057	2.80E−13	CAD	547261	−0.0011	0.0040	0.7910	Lifespan	610670
100	rs6494488	A	G	0.0382	0.0070	3.90E−08	CAD	547261	0.0074	0.0055	0.1811	Lifespan	637312
101	rs651821	T	C	−0.0692	0.0096	7.03E−13	CAD	547261	0.0224	0.0081	0.0055	Lifespan	640075
102	rs6544713	T	C	0.0492	0.0056	1.84E−18	CAD	547261	−0.0066	0.0041	0.1103	Lifespan	640181
103	rs6728861	A	G	0.1054	0.0089	1.29E−32	CAD	547261	−0.0079	0.0058	0.1699	Lifespan	638116
104	rs6743030	T	C	0.0565	0.0057	1.81E−23	CAD	547261	−0.0039	0.0039	0.3149	Lifespan	633075

105	rs6841581	A	G	0.0751	0.0074	5.18E−24	CAD	547261	−0.0127	0.0055	0.0220	Lifespan	638110
106	rs6905288	A	G	0.0386	0.0056	4.00E−12	CAD	547261	−0.0104	0.0040	0.0089	Lifespan	635385
107	rs6982502	T	C	−0.0498	0.0051	7.67E−23	CAD	547261	0.0100	0.0039	0.0093	Lifespan	640187
108	rs6984210	C	G	−0.0784	0.0115	1.04E−11	CAD	547261	0.0086	0.0087	0.3238	Lifespan	633977
109	rs7116641	T	G	−0.0314	0.0055	1.03E−08	CAD	547261	0.0068	0.0042	0.1047	Lifespan	637316
110	rs7145159	T	C	−0.0319	0.0051	3.60E−10	CAD	547261	0.0134	0.0039	0.0005	Lifespan	638118
111	rs71566846	T	C	0.0829	0.0114	3.24E−13	CAD	547261	−0.0004	0.0075	0.9552	Lifespan	640183
112	rs7188857	A	G	0.0485	0.0070	3.29E−12	CAD	547261	0.0013	0.0048	0.7881	Lifespan	635243
113	rs7199941	A	G	0.0367	0.0051	9.36E−13	CAD	547261	−0.0089	0.0039	0.0236	Lifespan	638108
114	rs7251815	T	G	0.0511	0.0069	1.44E−13	CAD	547261	−0.0066	0.0047	0.1583	Lifespan	640180
115	rs7256873	A	G	−0.0413	0.0075	3.32E−08	CAD	547261	0.0087	0.0051	0.0897	Lifespan	635243
116	rs72743461	A	C	−0.0576	0.0069	5.68E−17	CAD	547261	0.0023	0.0045	0.6091	Lifespan	639084
117	rs73079003	A	G	−0.0505	0.0090	1.81E−08	CAD	547261	0.0233	0.0060	0.0001	Lifespan	638083
118	rs7500448	A	G	0.0557	0.0068	1.61E−16	CAD	547261	−0.0018	0.0045	0.6897	Lifespan	635243
119	rs7617773	T	C	0.0355	0.0054	3.48E−11	CAD	547261	−0.0079	0.0041	0.0546	Lifespan	637310
120	rs7621025	T	C	−0.0454	0.0059	1.28E−14	CAD	547261	0.0110	0.0044	0.0128	Lifespan	640157
121	rs7678555	A	C	−0.0486	0.0063	1.23E−14	CAD	547261	0.0067	0.0043	0.1191	Lifespan	635243
122	rs7692387	A	G	−0.0643	0.0065	3.34E−23	CAD	547261	0.0020	0.0049	0.6816	Lifespan	640187
123	rs7695332	T	C	0.0469	0.0086	4.75E−08	CAD	547261	−0.0053	0.0064	0.4024	Lifespan	637314
124	rs7696431	T	G	0.0311	0.0056	3.09E−08	CAD	547261	−0.0023	0.0039	0.5553	Lifespan	638076
125	rs789294	C	G	−0.0642	0.0081	3.36E−15	CAD	547261	0.0027	0.0053	0.6137	Lifespan	637314
126	rs7926712	A	G	−0.0351	0.0061	9.41E−09	CAD	547261	0.0046	0.0042	0.2798	Lifespan	640173
127	rs8003602	T	C	−0.0539	0.0066	2.89E−16	CAD	547261	0.0056	0.0045	0.2104	Lifespan	640147
128	rs8080142	T	C	−0.0431	0.0078	3.10E−08	CAD	547261	0.0051	0.0052	0.3265	Lifespan	638068
129	rs867186	A	G	0.0573	0.0084	6.84E−12	CAD	547261	−0.0117	0.0067	0.0805	Lifespan	640167
130	rs885150	T	C	−0.0355	0.0058	7.86E−10	CAD	547261	0.0126	0.0044	0.0044	Lifespan	635243
131	rs9319428	A	G	0.0360	0.0055	5.36E−11	CAD	547261	−0.0066	0.0042	0.1148	Lifespan	640179
132	rs9337951	A	G	0.0543	0.0064	1.73E−17	CAD	547261	−0.0126	0.0042	0.0028	Lifespan	637310
133	rs944172	T	C	−0.0395	0.0057	3.59E−12	CAD	547261	0.0094	0.0043	0.0286	Lifespan	640181
134	rs9515203	T	C	0.0596	0.0060	3.89E−23	CAD	547261	−0.0104	0.0044	0.0187	Lifespan	637300
135	rs974819	T	C	0.0614	0.0055	1.12E−28	CAD	547261	−0.0082	0.0042	0.0548	Lifespan	640167
136	rs975722	A	G	−0.0283	0.0052	4.17E−08	CAD	547261	0.0118	0.0040	0.0028	Lifespan	640188
137	rs9892152	T	C	−0.0330	0.0050	6.28E−11	CAD	547261	0.0104	0.0039	0.0075	Lifespan	624084
138	rs9964304	A	C	−0.0382	0.0063	1.14E−09	CAD	547261	0.0041	0.0044	0.3476	Lifespan	626130
1	rs10401969	T	C	−0.1200	0.0140	7.70E−18	T2D	157384	−0.0006	0.0073	0.9369	Lifespan	640159
2	rs10471048	C	G	−0.0450	0.0078	8.00E−09	T2D	157384	0.0066	0.0041	0.1057	Lifespan	640179

3	rs1076902	T	C	0.0740	0.0120	1.00E−09	T2D	157384	0.0012	0.0063	0.8520	Lifespan	623208
4	rs10811660	A	G	−0.1800	0.0100	5.90E−69	T2D	157384	−0.0027	0.0051	0.6039	Lifespan	637314
5	rs10830963	C	G	−0.1000	0.0083	2.80E−36	T2D	157384	0.0040	0.0044	0.3638	Lifespan	635243
6	rs10842994	T	C	−0.0770	0.0094	2.00E−16	T2D	157384	0.0072	0.0049	0.1387	Lifespan	638090
7	rs10893829	T	C	0.0610	0.0110	7.00E−09	T2D	157384	0.0007	0.0055	0.9023	Lifespan	640177
8	rs10974438	A	C	−0.0590	0.0078	3.20E−14	T2D	157384	0.0041	0.0041	0.3103	Lifespan	635243
9	rs11048456	T	C	−0.0600	0.0086	2.60E−12	T2D	157384	0.0006	0.0045	0.8974	Lifespan	640185
10	rs1108646	A	G	0.0500	0.0080	4.40E−10	T2D	157384	−0.0048	0.0042	0.2544	Lifespan	637314
11	rs11257655	T	C	0.0950	0.0090	7.70E−26	T2D	157384	−0.0015	0.0048	0.7528	Lifespan	640182
12	rs112845979	T	C	−0.2700	0.0380	4.60E−12	T2D	157384	−0.0295	0.0212	0.1633	Lifespan	598386
13	rs114863656	T	C	0.1300	0.0200	1.40E−11	T2D	157384	0.0092	0.0103	0.3762	Lifespan	638116
14	rs115505614	T	C	0.1900	0.0180	3.80E−27	T2D	157384	0.0017	0.0091	0.8504	Lifespan	620104
15	rs11638890	T	G	0.0470	0.0079	3.40E−09	T2D	157384	−0.0006	0.0041	0.8749	Lifespan	635243
16	rs11688682	C	G	−0.0650	0.0088	1.60E−13	T2D	157384	−0.0034	0.0047	0.4642	Lifespan	637314
17	rs11819995	T	C	0.0550	0.0089	8.50E−10	T2D	157384	−0.0019	0.0046	0.6819	Lifespan	640189
18	rs1260326	T	C	−0.0670	0.0076	7.40E−19	T2D	157384	0.0015	0.0039	0.7100	Lifespan	640179
19	rs12611068	T	C	0.0550	0.0076	5.10E−13	T2D	157384	−0.0006	0.0040	0.8811	Lifespan	637314
20	rs12912777	T	C	0.0850	0.0120	3.30E−13	T2D	157384	−0.0006	0.0060	0.9209	Lifespan	637314
21	rs1320164	A	G	−0.0510	0.0074	5.50E−12	T2D	157384	0.0072	0.0039	0.0616	Lifespan	622318
22	rs13266634	T	C	−0.1200	0.0081	3.10E−53	T2D	157384	0.0018	0.0042	0.6742	Lifespan	640181
23	rs1359790	A	G	−0.0910	0.0083	6.50E−28	T2D	157384	−0.0022	0.0043	0.6077	Lifespan	635243
24	rs1426371	A	G	−0.0510	0.0086	3.50E−09	T2D	157384	0.0063	0.0045	0.1599	Lifespan	637314
25	rs146886108	T	C	−0.3900	0.0520	5.60E−14	T2D	149388	−0.0339	0.0262	0.1943	Lifespan	342756
26	rs1573090	T	G	0.0550	0.0076	6.10E−13	T2D	157384	0.0013	0.0039	0.7400	Lifespan	640157
27	rs17036160	T	C	−0.1200	0.0110	8.60E−26	T2D	157384	−0.0023	0.0060	0.7012	Lifespan	631445
28	rs17122772	C	G	−0.0520	0.0091	8.00E−09	T2D	157384	−0.0006	0.0047	0.8936	Lifespan	637314
29	rs17168486	T	C	0.0730	0.0098	7.50E−14	T2D	157384	−0.0047	0.0051	0.3559	Lifespan	640177
30	rs17772814	A	G	−0.0820	0.0150	4.00E−08	T2D	157384	0.0039	0.0078	0.6134	Lifespan	633312
31	rs17791513	A	G	0.1000	0.0150	8.10E−12	T2D	157384	−0.0091	0.0080	0.2585	Lifespan	638912
32	rs1800961	T	C	0.1800	0.0210	9.20E−18	T2D	157384	−0.0175	0.0110	0.1126	Lifespan	637676
33	rs2215383	T	C	−0.0710	0.0075	1.70E−21	T2D	157384	0.0028	0.0039	0.4750	Lifespan	638115
34	rs2237895	A	C	−0.1100	0.0076	9.90E−47	T2D	157384	−0.0024	0.0040	0.5484	Lifespan	637314
35	rs2238689	T	C	−0.0720	0.0076	1.40E−21	T2D	157384	0.0033	0.0040	0.4148	Lifespan	627210
36	rs2239526	A	G	0.0590	0.0085	3.00E−12	T2D	157073	−0.0051	0.0045	0.2552	Lifespan	613714
37	rs2258238	A	T	−0.1200	0.0120	1.90E−21	T2D	157384	0.0056	0.0063	0.3770	Lifespan	640161
38	rs2303700	T	C	0.0560	0.0081	4.70E−12	T2D	157384	−0.0026	0.0043	0.5469	Lifespan	612516

39	rs231360	T	C	0.0700	0.0077	1.80E−19	T2D	157384	−0.0011	0.0040	0.7742	Lifespan	635243
40	rs2494196	A	C	−0.0610	0.0083	2.20E−13	T2D	157384	0.0053	0.0043	0.2131	Lifespan	640187
41	rs2642596	T	C	0.0460	0.0075	1.00E−09	T2D	157384	−0.0014	0.0039	0.7121	Lifespan	637314
42	rs2648731	A	G	0.0510	0.0090	1.50E−08	T2D	157384	0.0051	0.0046	0.2719	Lifespan	640187
43	rs2661794	A	C	−0.0440	0.0076	9.60E−09	T2D	157384	−0.0017	0.0040	0.6740	Lifespan	635243
44	rs2747567	A	G	−0.0470	0.0078	1.30E−09	T2D	157384	0.0057	0.0040	0.1569	Lifespan	635243
45	rs2796441	A	G	−0.0800	0.0076	1.30E−25	T2D	157384	0.0062	0.0039	0.1160	Lifespan	635243
46	rs28429551	A	T	0.0790	0.0088	2.00E−19	T2D	157384	−0.0032	0.0048	0.4987	Lifespan	611107
47	rs28661116	A	G	0.0450	0.0075	1.50E−09	T2D	157384	−0.0017	0.0039	0.6562	Lifespan	640167
48	rs28678152	T	C	0.0790	0.0085	1.90E−20	T2D	157384	−0.0064	0.0045	0.1512	Lifespan	637314
49	rs2972144	A	G	−0.1000	0.0078	2.90E−40	T2D	157384	0.0074	0.0040	0.0647	Lifespan	640180
50	rs2982521	A	T	0.0440	0.0077	8.80E−09	T2D	157384	−0.0009	0.0040	0.8272	Lifespan	640186
51	rs3094515	T	C	−0.0470	0.0085	4.20E−08	T2D	157384	0.0031	0.0044	0.4807	Lifespan	638254
52	rs3212880	A	G	0.1400	0.0250	1.10E−08	T2D	157384	−0.0028	0.0127	0.8250	Lifespan	627263
53	rs329124	A	G	−0.0550	0.0075	1.70E−13	T2D	157384	0.0037	0.0039	0.3473	Lifespan	638107
54	rs340874	T	C	−0.0710	0.0075	4.80E−21	T2D	157384	−0.0070	0.0039	0.0721	Lifespan	638116
55	rs34584161	A	G	0.0510	0.0088	5.20E−09	T2D	157384	−0.0004	0.0046	0.9319	Lifespan	638875
56	rs34744311	T	C	−0.1200	0.0077	1.00E−57	T2D	157384	0.0041	0.0040	0.3126	Lifespan	628619
57	rs348330	A	G	−0.0520	0.0078	3.70E−11	T2D	157384	0.0008	0.0041	0.8461	Lifespan	635243
58	rs34872471	T	C	−0.3600	0.0082	1.00E−200	T2D	157384	0.0057	0.0043	0.1764	Lifespan	640174
59	rs35352848	T	C	0.0890	0.0092	5.40E−22	T2D	157384	−0.0025	0.0048	0.5980	Lifespan	638116
60	rs3757971	T	C	−0.0630	0.0078	8.20E−16	T2D	157384	−0.0009	0.0041	0.8201	Lifespan	623282
61	rs3768321	T	G	0.0750	0.0094	9.60E−16	T2D	157384	−0.0088	0.0049	0.0706	Lifespan	635243
62	rs3847343	A	G	0.0450	0.0078	1.10E−08	T2D	157384	−0.0042	0.0041	0.3064	Lifespan	637314
63	rs3934712	T	C	−0.0510	0.0092	3.20E−08	T2D	157384	0.0034	0.0047	0.4722	Lifespan	639123
64	rs4132228	T	C	−0.0680	0.0083	2.60E−16	T2D	157384	−0.0073	0.0042	0.0829	Lifespan	640171
65	rs4238013	T	C	−0.0700	0.0093	4.10E−14	T2D	157384	0.0051	0.0050	0.3011	Lifespan	637314
66	rs4457053	A	G	−0.0690	0.0081	1.50E−17	T2D	157384	0.0078	0.0042	0.0620	Lifespan	637314
67	rs4679370	T	C	−0.0470	0.0075	3.40E−10	T2D	157384	0.0051	0.0039	0.1860	Lifespan	638088
68	rs4686471	T	C	−0.0700	0.0077	1.10E−19	T2D	157384	−0.0010	0.0040	0.8088	Lifespan	640171
69	rs4709746	T	C	−0.0740	0.0110	3.50E−11	T2D	157384	0.0028	0.0058	0.6263	Lifespan	635243
70	rs4865436	C	G	−0.0580	0.0088	3.50E−11	T2D	157384	0.0032	0.0047	0.4883	Lifespan	629778
71	rs4865796	A	G	0.0610	0.0081	6.70E−14	T2D	157384	−0.0050	0.0042	0.2284	Lifespan	640132
72	rs4923543	A	G	0.0430	0.0079	4.50E−08	T2D	157384	0.0031	0.0041	0.4463	Lifespan	640183
73	rs4929965	A	G	0.0700	0.0078	1.60E−19	T2D	157384	−0.0025	0.0040	0.5358	Lifespan	630063
74	rs4976033	A	G	−0.0470	0.0077	1.00E−09	T2D	157384	0.0072	0.0040	0.0755	Lifespan	637314

75	rs515071	A	G	−0.0970	0.0088	4.90E−28	T2D	157384	0.0053	0.0045	0.2367	Lifespan	638089
76	rs5215	T	C	−0.0790	0.0077	9.40E−25	T2D	157384	0.0044	0.0040	0.2677	Lifespan	640183
77	rs56187241	T	C	0.1300	0.0190	7.30E−12	T2D	157384	−0.0116	0.0101	0.2489	Lifespan	628152
78	rs58432198	T	C	−0.0730	0.0120	1.40E−09	T2D	157073	0.0024	0.0061	0.6893	Lifespan	635243
79	rs591291	T	C	0.0580	0.0081	7.30E−13	T2D	157384	−0.0001	0.0042	0.9819	Lifespan	624042
80	rs59944054	A	G	0.0480	0.0088	4.60E−08	T2D	157384	0.0083	0.0046	0.0714	Lifespan	637314
81	rs61462211	C	G	0.0680	0.0082	1.00E−16	T2D	157384	0.0010	0.0043	0.8153	Lifespan	623282
82	rs62563593	A	G	−0.0480	0.0076	3.20E−10	T2D	157384	0.0055	0.0039	0.1604	Lifespan	640185
83	rs6459733	C	G	−0.0620	0.0080	1.00E−14	T2D	157384	0.0063	0.0041	0.1275	Lifespan	640169
84	rs6474360	A	G	−0.0850	0.0160	4.90E−08	T2D	157384	0.0052	0.0081	0.5192	Lifespan	637314
85	rs6600191	T	C	0.0650	0.0098	3.80E−11	T2D	157384	0.0019	0.0050	0.6987	Lifespan	626151
86	rs6770420	A	G	−0.0750	0.0077	2.60E−22	T2D	157384	0.0011	0.0040	0.7810	Lifespan	640182
87	rs6780171	A	T	0.1200	0.0080	2.40E−51	T2D	157384	−0.0051	0.0042	0.2171	Lifespan	640161
88	rs6813195	T	C	−0.0630	0.0084	7.00E−14	T2D	157384	−0.0003	0.0045	0.9535	Lifespan	640186
89	rs6885132	C	G	0.0950	0.0130	6.60E−14	T2D	157384	−0.0060	0.0066	0.3619	Lifespan	637314
90	rs7015203	T	C	0.0440	0.0075	5.20E−09	T2D	157384	−0.0022	0.0039	0.5707	Lifespan	637314
91	rs7018475	T	G	−0.1200	0.0085	4.10E−45	T2D	157384	−0.0030	0.0046	0.5141	Lifespan	597911
92	rs703967	A	C	0.0800	0.0075	3.00E−26	T2D	157384	−0.0029	0.0039	0.4537	Lifespan	640183
93	rs719727	A	G	0.0740	0.0087	2.40E−17	T2D	157384	0.0054	0.0045	0.2238	Lifespan	640187
94	rs72631105	A	G	0.0580	0.0098	3.70E−09	T2D	157384	−0.0004	0.0051	0.9306	Lifespan	635243
95	rs72802342	A	C	−0.1600	0.0140	2.70E−29	T2D	157384	−0.0071	0.0075	0.3424	Lifespan	635243
96	rs738408	T	C	0.0620	0.0089	3.00E−12	T2D	157384	0.0054	0.0047	0.2476	Lifespan	638083
97	rs73875816	T	C	0.0710	0.0110	2.00E−11	T2D	157384	0.0077	0.0055	0.1634	Lifespan	640154
98	rs73883375	A	G	0.0850	0.0130	1.40E−10	T2D	157384	0.0008	0.0067	0.9021	Lifespan	640181
99	rs73927890	T	C	−0.0530	0.0085	3.90E−10	T2D	157384	0.0051	0.0044	0.2487	Lifespan	640185
100	rs7451008	T	C	−0.1500	0.0083	7.00E−76	T2D	157384	0.0004	0.0044	0.9200	Lifespan	640142
101	rs7579654	T	C	−0.0920	0.0150	1.80E−09	T2D	157384	−0.0066	0.0079	0.4013	Lifespan	640187
102	rs76093749	T	C	0.1100	0.0190	3.20E−08	T2D	157384	0.0116	0.0104	0.2654	Lifespan	626245
103	rs76223293	C	G	0.0550	0.0096	1.30E−08	T2D	157384	−0.0071	0.0050	0.1527	Lifespan	639103
104	rs7640397	T	C	0.0460	0.0075	5.60E−10	T2D	157384	0.0018	0.0039	0.6511	Lifespan	638116
105	rs7660000	T	C	−0.0460	0.0083	3.70E−08	T2D	157384	0.0005	0.0043	0.9109	Lifespan	640174
106	rs76895963	T	G	0.5200	0.0310	1.30E−64	T2D	157384	−0.0051	0.0155	0.7431	Lifespan	623075
107	rs77101426	A	G	−0.1600	0.0150	5.50E−28	T2D	157384	0.0047	0.0075	0.5307	Lifespan	638097
108	rs77464186	A	C	0.1300	0.0100	9.00E−37	T2D	157384	0.0052	0.0053	0.3284	Lifespan	640178
109	rs79221399	A	C	−0.0820	0.0140	6.50E−09	T2D	157384	0.0009	0.0072	0.8992	Lifespan	638921
110	rs8017808	T	G	−0.0480	0.0087	3.70E−08	T2D	157384	−0.0038	0.0045	0.3970	Lifespan	640167

111	rs8063007	A	C	−0.0580	0.0110	2.70E−08	T2D	157384	0.0034	0.0055	0.5309	Lifespan	640185
112	rs849135	A	G	−0.1000	0.0075	7.20E−41	T2D	157384	0.0048	0.0039	0.2172	Lifespan	640167
113	rs858519	T	C	0.0410	0.0075	3.70E−08	T2D	157384	0.0008	0.0039	0.8385	Lifespan	626548
114	rs867489	T	C	−0.0560	0.0075	1.00E−13	T2D	157384	0.0043	0.0039	0.2710	Lifespan	635243
115	rs878521	A	G	0.0620	0.0087	8.70E−13	T2D	157384	−0.0056	0.0045	0.2087	Lifespan	640162
116	rs9275152	T	C	−0.1200	0.0130	1.20E−21	T2D	157074	0.0102	0.0072	0.1588	Lifespan	565394
117	rs9379084	A	G	−0.1200	0.0120	3.80E−23	T2D	157384	−0.0109	0.0064	0.0867	Lifespan	626548
118	rs9394969	T	G	0.0440	0.0075	3.80E−09	T2D	157384	−0.0003	0.0039	0.9355	Lifespan	638203
119	rs963740	A	T	0.0460	0.0082	1.40E−08	T2D	157384	−0.0038	0.0042	0.3715	Lifespan	636848
1	rs1021363	A	G	0.0300	0.0045	2.29E−11	Depression	500199	−0.0071	0.0040	0.0782	Lifespan	638108
2	rs10235664	T	C	0.0270	0.0049	4.68E−08	Depression	500199	−0.0073	0.0045	0.1018	Lifespan	638071
3	rs10501696	A	G	0.0295	0.0044	2.89E−11	Depression	500199	−0.0079	0.0040	0.0478	Lifespan	637314
4	rs10913112	T	C	−0.0262	0.0045	4.53E−09	Depression	500199	0.0047	0.0041	0.2424	Lifespan	640187
5	rs12631196	A	G	0.0241	0.0044	3.28E−08	Depression	500199	−0.0001	0.0039	0.9868	Lifespan	637314
6	rs12723839	A	T	0.0463	0.0076	9.30E−10	Depression	500199	0.0015	0.0068	0.8208	Lifespan	640188
7	rs12919291	C	G	0.0327	0.0055	3.09E−09	Depression	500199	0.0028	0.0050	0.5706	Lifespan	638104
8	rs13037326	T	C	0.0310	0.0049	2.40E−10	Depression	500199	−0.0129	0.0044	0.0032	Lifespan	640183
9	rs1931388	A	G	0.0295	0.0044	1.68E−11	Depression	500199	−0.0022	0.0039	0.5802	Lifespan	640155
10	rs1950829	A	G	0.0297	0.0043	4.74E−12	Depression	500199	−0.0059	0.0038	0.1235	Lifespan	640182
11	rs198457	T	C	−0.0315	0.0056	1.90E−08	Depression	500199	−0.0105	0.0050	0.0370	Lifespan	637314
12	rs2111592	A	G	0.0263	0.0046	1.35E−08	Depression	500199	−0.0043	0.0041	0.3044	Lifespan	638102
13	rs2214123	A	G	0.0261	0.0045	8.56E−09	Depression	500199	−0.0041	0.0041	0.3118	Lifespan	635243
14	rs2418449	T	C	0.0281	0.0048	4.25E−09	Depression	500199	0.0044	0.0043	0.3102	Lifespan	626155
15	rs2568958	A	G	0.0382	0.0044	2.90E−18	Depression	500199	−0.0093	0.0039	0.0182	Lifespan	640053
16	rs28541419	C	G	0.0292	0.0052	1.76E−08	Depression	500199	0.0016	0.0047	0.7319	Lifespan	635243
17	rs30266	A	G	0.0366	0.0046	1.43E−15	Depression	500199	−0.0015	0.0041	0.7196	Lifespan	640173
18	rs3099439	T	C	−0.0241	0.0043	2.78E−08	Depression	500199	−0.0055	0.0039	0.1613	Lifespan	640159
19	rs3807865	A	G	0.0310	0.0044	1.09E−12	Depression	500199	−0.0023	0.0039	0.5644	Lifespan	638074
20	rs4141983	T	C	0.0264	0.0046	9.69E−09	Depression	500199	0.0018	0.0042	0.6605	Lifespan	640187
21	rs4799949	T	C	−0.0292	0.0046	1.40E−10	Depression	500199	0.0067	0.0041	0.1057	Lifespan	631469
22	rs4936275	T	C	0.0278	0.0044	3.35E−10	Depression	500199	−0.0121	0.0040	0.0023	Lifespan	640187
23	rs508502	T	C	−0.0264	0.0048	3.56E−08	Depression	500199	0.0114	0.0043	0.0075	Lifespan	633977
24	rs55658856	A	G	−0.0781	0.0132	3.13E−09	Depression	500199	0.0030	0.0119	0.8030	Lifespan	637675
25	rs59082935	T	C	0.0363	0.0066	3.07E−08	Depression	500199	0.0027	0.0059	0.6502	Lifespan	621211
26	rs61914045	A	G	0.0309	0.0054	7.96E−09	Depression	500199	−0.0036	0.0048	0.4546	Lifespan	640152
27	rs62535714	A	G	0.0339	0.0058	4.69E−09	Depression	500199	0.0052	0.0052	0.3146	Lifespan	640183

28	rs66511648	T	C	−0.0297	0.0048	6.03E−10	Depression	500199	−0.0068	0.0043	0.1140	Lifespan	635243
29	rs6656912	T	C	−0.0252	0.0043	6.50E−09	Depression	500199	0.0011	0.0039	0.7875	Lifespan	640187
30	rs7152906	T	C	−0.0258	0.0043	1.87E−09	Depression	500199	0.0053	0.0038	0.1702	Lifespan	640179
31	rs7241572	A	G	0.0323	0.0054	2.43E−09	Depression	500199	−0.0062	0.0049	0.1995	Lifespan	637314
32	rs72948506	A	G	0.0265	0.0047	1.72E−08	Depression	500199	−0.0047	0.0042	0.2672	Lifespan	631474
33	rs754287	A	T	−0.0289	0.0045	1.31E−10	Depression	500199	−0.0012	0.0040	0.7631	Lifespan	638110
34	rs7551758	T	G	−0.0283	0.0043	5.11E−11	Depression	500199	0.0097	0.0039	0.0121	Lifespan	638091
35	rs76954012	A	T	0.0412	0.0074	2.41E−08	Depression	500199	−0.0102	0.0067	0.1274	Lifespan	640145
36	rs7725715	A	G	0.0290	0.0043	1.61E−11	Depression	500199	0.0023	0.0039	0.5541	Lifespan	638110
37	rs843812	A	G	0.0248	0.0044	1.41E−08	Depression	500199	0.0049	0.0039	0.2165	Lifespan	637314
38	rs9364755	A	G	−0.0283	0.0051	3.49E−08	Depression	500199	0.0029	0.0046	0.5243	Lifespan	631021
39	rs9408078	T	G	0.0367	0.0069	1.03E−07	Depression	500199	−0.0094	0.0062	0.1295	Lifespan	640189
40	rs9529218	T	C	−0.0340	0.0054	2.23E−10	Depression	500199	0.0053	0.0048	0.2701	Lifespan	633977
41	rs9536381	T	C	0.0255	0.0046	2.62E−08	Depression	500199	−0.0016	0.0041	0.6908	Lifespan	638884
42	rs970185	A	T	0.0276	0.0044	2.83E−10	Depression	500199	−0.0086	0.0039	0.0278	Lifespan	638078
43	rs9831648	T	G	−0.0292	0.0052	1.59E−08	Depression	500199	0.0089	0.0046	0.0523	Lifespan	640187

Table S5. Mediation MR results of short sleep duration on BMI, heart failure, atrial fibrillation, any ischemic stroke, schizophrenia and bipolar disorder

MR Method	Number of IVs	*F*	*β* (95%CI)	*P*
**Short sleep duration on BMI**				
IVW	9	39	0.24 (0.01 to 0.47)	0.04
Weighted median			0.28 (0.02 to 0.54)	0.03
MR-Egger			−0.20 (−1.15 to 0.75)	0.69
Weighted mode			0.37 (−0.01 to 0.75)	0.09
MR.RAPS			0.23 (0.01 to 0.46)	0.04
**Short sleep duration on Heart failure**			**OR (95%CI)**	
IVW	23	38	2.11 (1.09 to 4.07)	0.03
Weighted median			1.43 (0.64 to 3.19)	0.38
MR-Egger			29.33 (1.46 to 589.59)	0.04
Weighted mode			1.20 (0.33 to 4.36)	0.78
MR.RAPS			1.84 (1.03 to 3.28)	0.04
**Short sleep duration on Atrial fibrillation**			**OR (95%CI)**	
IVW	56	38	1.56 (0.89 to 2.72)	0.12
Weighted median			1.26 (0.60 to 2.65)	0.54
MR-Egger			1.59 (0.14 to 18.16)	0.71
Weighted mode			1.08 (0.32 to 3.62)	0.91
MR.RAPS			1.55 (0.83 to 2.87)	0.17
**Short sleep duration on Any ischemic stroke**			**OR (95%CI)**	
IVW	64	38	1.41 (0.70 to 2.84)	0.34
Weighted median			1.30 (0.52 to 3.27)	0.58
MR-Egger			3.08 (0.11 to 86.22)	0.52
Weighted mode			1.16 (0.21 to 6.40)	0.86
MR.RAPS			1.38 (0.66 to 2.87)	0.39
**Short sleep duration on Schizophrenia**			**OR (95%CI)**	
IVW	16	36	1.21 (0.31 to 4.68)	0.79
Weighted median			0.55 (0.15 to 1.96)	0.35
MR-Egger			0.05 (7.76×10^{-5} to 30.24)	0.37
Weighted mode			0.32 (0.04 to 2.31)	0.28
MR.RAPS			0.96 (0.22 to 4.28)	0.96
**Short sleep duration on Bipolar disorder**			**OR (95%CI)**	
IVW	20	38	1.63 (0.58 to 4.34)	0.25
Weighted median			1.58 (0.72 to 3.70)	0.37
MR-Egger			10.20 (0.26 to 401.99)	0.23
Weighted mode			1.50 (0.28 to 7.96)	0.64
MR.RAPS			1.39 (0.64 to 3.01)	0.40
